# miR-449a mediated repression of the cell cycle machinery prevents neuronal apoptosis

**DOI:** 10.1016/j.jbc.2024.107698

**Published:** 2024-08-22

**Authors:** Monika Chauhan, Komal Singh, Chen Chongtham, Aneeshkumar A.G., Pushkar Sharma

**Affiliations:** 1 Eukaryotic Gene Expression Laboratory, National Institute of Immunology, New Delhi, India; 2 Molecular Genetics Laboratory, National Institute of Immunology, New Delhi, India

**Keywords:** signaling, Alzheimer's disease, cell cycle, neuronal apoptosis, microRNA

## Abstract

Aberrant activation of the cell cycle of terminally differentiated neurons results in their apoptosis and is known to contribute to neuronal loss in various neurodegenerative disorders like Alzheimer's Disease. However, the mechanisms that regulate cell cycle–related neuronal apoptosis are poorly understood. We identified several miRNA that are dysregulated in neurons from a transgenic APP/PS1 mouse model for AD (TgAD). Several of these miRNA are known to and/or are predicted to target cell cycle–related genes. Detailed investigation on miR-449a revealed the following: a, it promotes neuronal differentiation by suppressing the neuronal cell cycle; b, its expression in cortical neurons was impaired in response to amyloid peptide Aβ_42_; c, loss of its expression resulted in aberrant activation of the cell cycle leading to apoptosis. miR-449a may prevent cell cycle–related neuronal apoptosis by targeting cyclin D1 and protein phosphatase CDC25A, which are important for G1-S transition. Importantly, the lentiviral-mediated delivery of miR-449a in TgAD mouse brain significantly reverted the defects in learning and memory, which are associated with AD.

Neural precursor cells differentiate into neurons by exiting the cell cycle (1) and the cell cycle of terminally differentiated neurons remains arrested for the remainder of their life span (2). Therefore, the machinery involved in cell cycle progression, which constitutes key regulatory elements like cyclins, cyclin-dependent kinases (CDKs), and CDK inhibitors, needs tight regulation (3). These and other elements form a network that works in an orchestrated way to promote the progression of the cell cycle. For instance, the expression of D-type cyclins like cyclin D1 is induced by signaling pathways stimulated by mitogenic signals (4). Cyclin D associates with CDK4/6 and activates it, which in turn phosphorylates retinoblastoma (Rb) or other related proteins. In its unphosphorylated state, Rb proteins keep the transcription factor E2F sequestered and Rb-phosphorylation promotes dissociation of E2F and transcriptional activation. E2F is involved in the transcription of S-phase cyclin E, which activates CDK2 to promote S-phase progression (5, 6). CDKs are regulated by reversible phosphorylation; CAK phosphorylation of the activation loop of CDKs (at T160 in the case of CDK1) activates them whereas phosphorylation by Myt1 and Wee1 inhibits their activity. Protein phosphatase CDC25 and its isoforms dephosphorylate the Myt1- and Wee1-phosphorylated sites (T14 and Y15 in CDK1) and prevent activation of CDKs (7). There is substantial evidence which suggests that neurons can re-enter the cell cycle and undergo DNA replication in response to neurotoxic insults like DNA-damage and Aβ_42_ amyloid peptide (8, 9, 10). Rb knockout causes aberrant cell cycle re-entry of neurons *via* E2F1 (11), suggesting that it is critical for neuronal cell cycle to remain suppressed.

However, aberrant cell cycle re-entry does not culminate in mitosis; instead, it results in neuronal apoptosis and neuronal loss in the cortex (12). The expression and the activity of cell cycle regulators like the ones mentioned above is modulated, which contributes to the process of cell cycle–related neuronal apoptosis (CRNA) (13, 14, 15). Aβ_42_ generated during Alzheimer’s disease (AD), which forms oligomers and is a major constituent of plaques, is also known to cause aberrant cell cycle re-entry of cortical neurons, a commonly observed phenomenon in AD animal models (9, 16, 17, 18, 19, 20) and in the brain of AD patients.

miRNA are ∼22 nucleotide small noncoding RNAs that function by typically binding to the 3′-UTR of the target mRNA (21) and regulate the expression of the target by facilitating mRNA degradation or its translational repression. miRNA can regulate neuronal proliferation, differentiation, as well as apoptosis (22, 23, 24, 25, 26). While independent studies have indicated that several miRNA clusters are involved in the regulation of cell cycle–related genes (27, 28, 29), it still remains unclear if they contribute to neuronal differentiation.

MicroRNA such as miR-34a, miR-17-5p, miR-16, miR-449a, have been reported previously to be implicated in the process of cell cycle regulation (29). While altered expression of some of these cell cycle–related miRNA has been reported in neurodegenerative disorders (30, 31, 32, 33), their correlation with the neuronal cell cycle regulation and neuronal loss has remained unknown. RNA sequencing reported here revealed the identity of several miRNA that exhibit significantly altered expression in cortical neurons of a mouse model for AD-APP/PS1 (TgAD). The role of miR-449a in CRNA was investigated in detail. miR-449a belongs to *miR-449* cluster, which is located in the second intron of *cdc20b* gene on chromosome 5 (34, 35). It is highly expressed in the mucocilliary epithelia of lungs (36, 37). In the brain, miR-449a is expressed during the proliferative phase of embryonic neurogenesis (38) and in adult brain choroid plexus (39). It is also essential for the production of intermediate progenitors during cortical development (40, 41). The tumor suppressor role of miR-449a has been extensively studied in dividing cells in various types of cancers (42, 43, 44, 45, 46, 47). miR-449a has been shown to target cyclin D1 and prevent proliferation of cancer cells (45, 48).

We have found that miRNA-449a is downregulated upon Aβ_42_ treatment of neurons. It promoted neuronal differentiation by suppressing the cell cycle and prevents neuronal apoptosis by impairing aberrant activation of the cell cycle induced by Aβ_42_. Furthermore, miR-449a overexpression prevented several cognitive defects observed in an AD animal model.

## Results

### Identification of miRNA aberrantly regulated by Aβ_42_ in cortical neurons

We were interested in deciphering mechanisms involved in the regulation of neuronal cell cycle during CRNA observed in AD, which is dependent on aberrant expression of genes involved in cell cycle regulation. Given that miRNA regulates gene expression by post-transcriptional gene silencing and have been implicated in neuronal differentiation (23, 38, 49, 50) as well as regulation of cell cycle–related genes in cell division (51), we wanted to identify miRNA that may be dysregulated in AD and in turn may contribute to CRNA. It is well-known that aberrant generation and oligomerization of Aβ_42_ is one of the major contributors to plaque formation as well as neuronal cell death in AD. Aβ_42_ also contributes to CRNA in cortical neurons (9, 52, 53). In order to identify the miRNA that are aberrantly expressed in response to Aβ_42_, we used cortical neurons from APP/PS1 (TgAD) transgenic mice, a routinely used animal model for AD that expresses high amounts of Aβ_42_ that constitute amyloid plaques (54, 55, 56). Previous studies indicated that cortical neurons from these animals exhibit CRNA (14, 52, 57, 58) and suffer from several cognitive defects that are also observed in AD patients (59).

**Figure 1:**
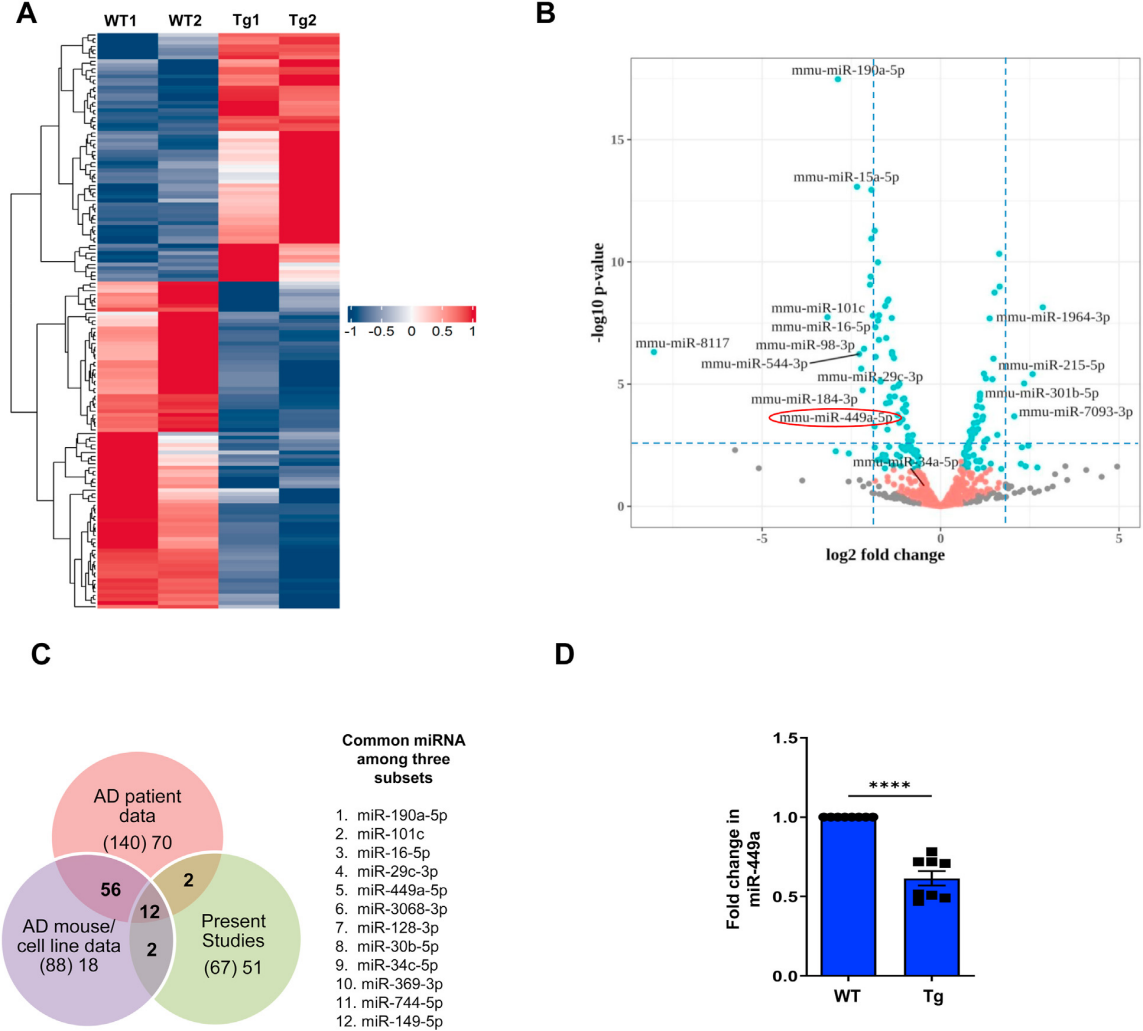
**Identification of miRNA dysregulated in response to Aβ**_**42**_**neurotoxicity.** Cortical neurons from WT and TgAD mice were cultured; total RNA was isolated and RNA sequencing was performed. *A*, heat-map displaying normalized read counts of upregulated or downregulated miRNA in TgAD mice compared to WT controls. *B*, volcano plot (log_2_ fold-change *versus* −log_10_*p*-value) indicating some of the dysregulated miRNAs (Dataset S1, log_2_ Fold Change >2 and *p*adj < 0.01). miR-449a, which is studied in detail, in the present study is encircled. *C*, Venn diagram showing the overlap between the miRNAs identified in this study (with stringent cutoffs) and previous reports. Twelve of the miRNA that were significantly altered in TgAD neurons in the present study were previously reported to be aberrantly expressed in human AD brain and AD mouse brain/AD cell culture models. *D*, cortical neurons were cultured from WT or APP/PS1 transgenic (TgAD) mice, and qRT-PCR was performed as mentioned above and fold change in miRNA-449a expression was determined (Mean ± SEM, ∗∗∗∗*p* < 0.0001, two tailed paired *t* test, N = 8).

The above mentioned APP/PS1 mice—hereafter referred to as TgAD mice—were used for present studies exhibited previously reported thioflavin-stained amyloid plaques (Fig. S5) and cognitive defects. First, small-RNAseq was performed on RNA extracted from WT and TgAD mice and data were analyzed using the pipeline indicated in Fig. S1*A*. The analysis identified 693 miRNAs (Dataset S1), out of which 151 were differentially expressed in TgAD neurons (87 downregulated and 64 upregulated) (Fig. 1*A*) when differential expression cutoffs used were log2 fold change >0.6 and *p*adj <0.1 (Fig. 1*B* and Dataset S1).

It was interesting to note that 19 of these miRNA (Fig. S1*B* and Dataset S3) have been reported to exhibit aberrant expression in either AD patients (60, 61, 62, 63, 64) and/or other related studies performed using AD animal or cell culture models (30, 65, 66, 67, 68, 69). A more stringent cutoff of log_2_ fold change >1 (fold change >2, adjusted *p*-value < 0.01, Fig. 1*B* and Dataset S1) revealed 67 (45 down and 22 upregulated) significantly altered miRNA; 12 of these miRNA were altered in the previous studies mentioned above (Fig. 1*C* and Dataset S3). Subsequently, we used *in silico* analysis to predict the ones that had the potential to target cell cycle–related genes using three independent algorithms miRDB (70), TargetScan (https://www.targetscan.org/vert_80/), RNA22 (71). These studies predicted several cell cycle–related genes to be putative targets of these miRNAs; some of these have also been experimentally validated. miR-449a emerged as an interesting candidate for further investigations as it has been reported to be expressed aberrantly in the cerebrospinal fluid (CSF) of AD patients (72, 73) and for other reasons that are discussed below in more detail. The downregulation of miR-449a in TgAD neurons was confirmed by quantitative RT-PCR from three independent biological replicates (Fig. 1*D*, discussed later). One of the major reasons for focusing on this miRNA was that it was predicted to target key G1-S transition regulators of the cell cycle like CDC25A and cyclin D1 by at least two of the algorithms (Fig. S2*A*, Table S1 and Dataset S4, discussed below). The entry into the cell cycle from terminally differentiated Go state first occurs *via* G1-S phase, and CDC25A and cyclin D1 play a critical role in this process, which made investigation of the role of miR-449a in CRNA very pertinent. Moreover, as indicated above, miR-449a is expressed during the proliferative phase of embryonic neurogenesis (38) and in adult brain choroid plexus (39) and is also essential for the production of intermediate progenitors during cortical development (40, 41). The tumor suppressor role of miR-449a (42, 43, 44, 45, 46, 47), which it may play by targeting cell cycle proteins like cyclin D1 (45, 48), is suggestive of its involvement in cell cycle regulation has been extensively studied in dividing cells in various types of cancers. In addition, miR-449a is aberrantly expressed in cerebrospinal fluid of AD patients (72, 73). Therefore, detailed investigations directed on miR-449a that was downregulated in TgAD neurons (Fig. 1, *B–D*) were carried out.

### miR-449a regulates neuronal differentiation by suppressing the neuronal cell cycle

**Figure 2:**
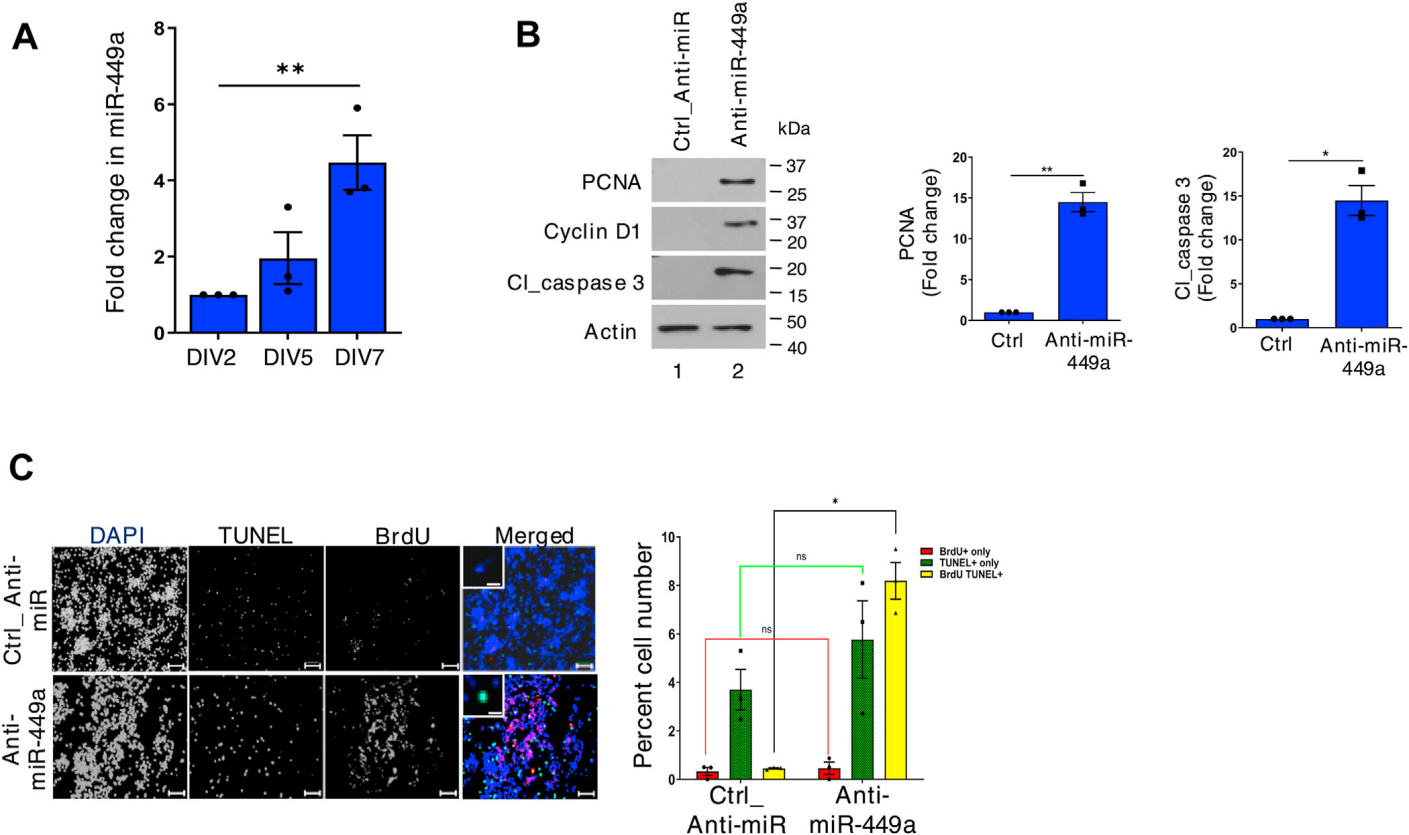
**miR-449a suppresses neuronal cell cycle during differentiation.***A*, neurons derived from rat embryo cortex were seeded and allowed to differentiate for 2, 5, and 7 days *in vitro* (DIV). qRT-PCR was performed to detect miR-449a expression and fold change with respect to its expression on day 2 was determined. Data represent mean ± SEM, ∗∗*p* < 0.01, One-way ANOVA with Tukey’s *post hoc* test, N = 3. *B*, rat cortical neurons transfected with antagomir for miR-449a (*B*) or a control antagomir. After 48 h, lysates were prepared and subjected to immunoblotting with antibodies against PCNA, cyclin D1, and cl_caspase 3. *Right panel*, densitometry for indicated proteins was performed and fold change in the expression after normalization with respect to actin was determined. Data represent mean ± SEM, ∗*p* < 0.05, ∗∗*p* < 0.01, two tailed paired *t* test, N = 3. *C*, rat cortical neurons were transfected with anti-miR-449a or a control inhibitor for 48 h followed by incubation with BrdU. Subsequently, immunofluorescence and TUNEL assays were performed to detect BrdU incorporation or apoptosis (*green*), respectively. Scale bar represents 100 μm (main image), 10 μm (inset). *Right panel*, % cells that were only stained with BrdU^+^, TUNEL^+^ (*green*), or both BrdU^+^/TUNEL^+^ (*yellow*) was determined (mean ± SEM, ∗*p* < 0.05, ns-not significant, two tailed paired *t* test, N = 3).

One of the major objectives of the present study was to decipher the connection between cell cycle and death of neurons. Therefore, we first investigated if miR-449a regulates the cell cycle during neuronal differentiation. For this purpose, primary cortical neurons were allowed to differentiate for 7 days *in vitro*; quantitative RT-PCR (qRT-PCR) revealed that the levels of both miRNA significantly increased with differentiation (Fig. 2*A*).

In the light of these observations, it was pertinent to test if miR-449a regulated the cell cycle during neuronal differentiation, which was done by assessing the levels of cyclin D1 (early G1 marker) and proliferating cell nuclear antigen (PCNA) (S-phase marker) that decrease upon differentiation of neurons as they exit the cell cycle (52). Rat cortical neurons were transfected with antagomiRs (anti-miR) to inhibit the expression of this miRNA, qRT-PCR revealed efficient depletion of miR-449a (Fig. S3*A*). Western blotting revealed that cyclin D1 and PCNA that were expressed at very low levels or were almost absent in differentiated neurons (52, 53) (Fig. 2*B* lane 1) were elevated upon miR-449a inhibition (Fig. 2*B*). Simultaneously, apoptotic marker cleaved caspase 3 was also upregulated upon inhibiting miR-449a expression. Furthermore, BrdU-labeling and terminal deoxynucleotidyl transferase dUTP nick end labeling (TUNEL) assays were performed, which are indicative of DNA replication and apoptosis, respectively. A significant increase in the fraction of cells which were both BrdU+ and TUNEL+ was observed upon miR-449a inhibition (Fig. 2*C*), which suggested that there was an increase in the reactivation of cell cycle and apoptosis of these cells. Collectively, these data indicated that miR-449a keeps the neuronal cell cycle suppressed, which is critical for maintaining the state of differentiation failing in which neurons undergo apoptosis.

### miR-449a is downregulated in response to Aβ_42_, which results in CRNA

**Figure 3:**
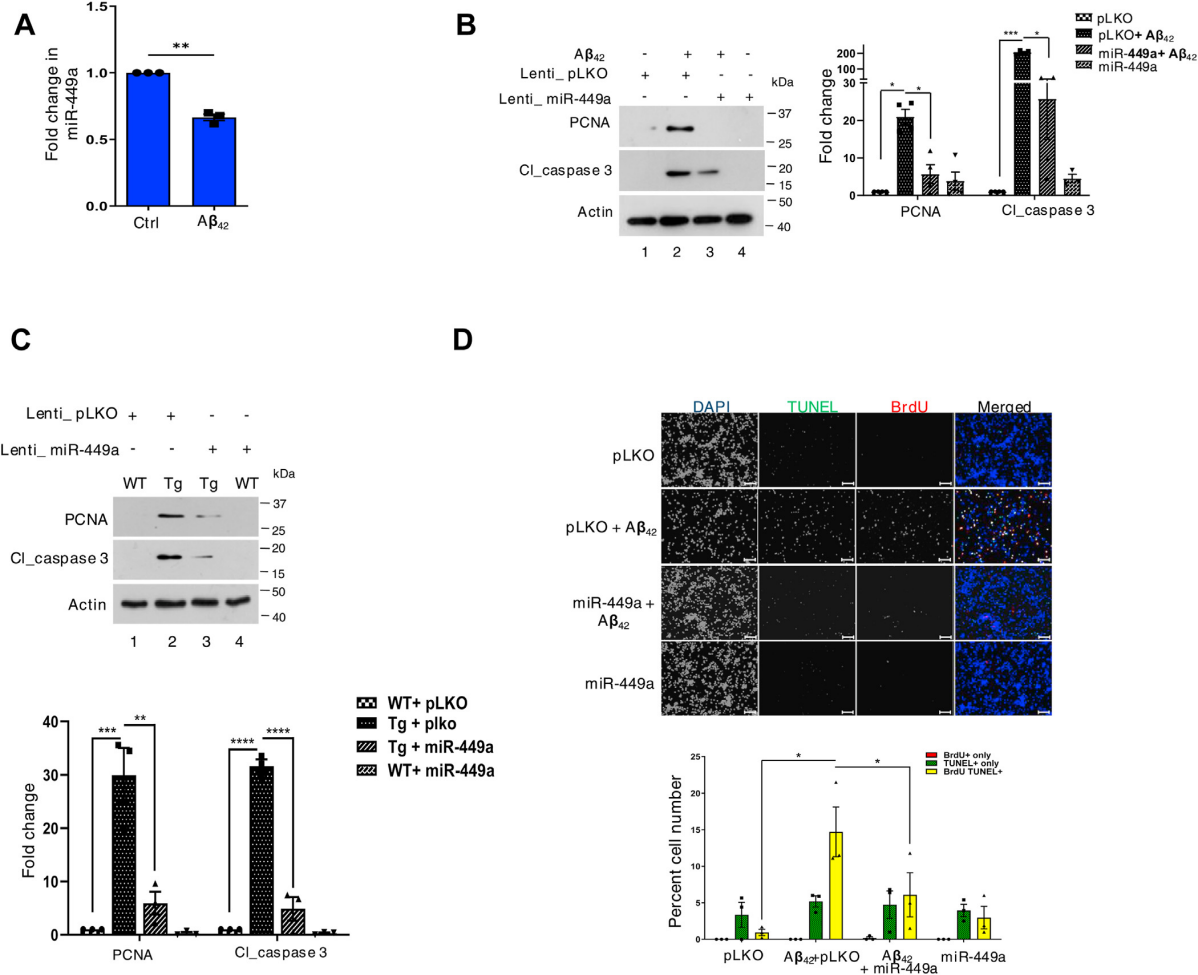
**Impaired miR-449a expression in AD may contribute to cell cycle–related neuronal apoptosis.***A*, rat cortical neurons were left untreated or treated with Aβ_42_ for 48 h. Total RNA was isolated and qRT-PCR was performed to determine the levels of miR-449a and fold change in miRNA expression with respect to untreated neurons is provided (mean ± SEM, ∗∗*p* < 0.01, two tailed paired *t* test, N = 3). *B*, rat cortical neurons were transduced with lentivirus expressing miR-449a or control lentivirus (pLKO) followed by treatment with Aβ_42_ for 48 h. Subsequently, Western blotting was done with indicated antibodies. *Right panel*, PCNA and cl_caspase 3 expression levels were quantified by densitometry (Data represent mean ± SEM, ∗*p* < 0.05, ∗∗∗*p* < 0.001, two-way ANOVA with Tukey’s multiple comparison, N = 4). *C*, cortical neurons cultured from WT or TgAD mice were transduced with miR-449a or control lentivirus (pLKO). After 48 h, cell lysates were prepared and Western blotting was performed for PCNA, cl_caspase 3, or actin. *Bottom panel*, densitometry for indicated proteins normalized with respect to actin (Data represent mean ± SEM, ∗∗*p* < 0.01, ∗∗∗*p* < 0.001, ∗∗∗∗*p* < 0.0001 by two-way ANOVA with Tukey’s multiple comparison, N = 3). *D*, rat cortical neurons were transduced with lentivirus expressing miR-449a or control virus (pLKO) and treated with Aβ_42_ for 48 h, followed by incubation with BrdU. Subsequently, immunofluorescence and TUNEL assay were performed to detect BrdU incorporation or apoptosis, respectively. Scale bar represents 100 μm. *Bottom panel*, % cells that were only BrdU+ (*red*), TUNEL+ (*green*), or both BrdU+/TUNEL+ (*yellow*) were determined (mean ± SEM, ∗*p* < 0.05, two tailed paired *t* test, N = 3).

There was ∼50% decrease in miR-449a expression in TgAD neurons in comparison to the WT counterparts (Fig. 1*B* and Dataset S2), which was validated by qRT-PCR performed on TgAD neurons (Fig. 1*D*). We further assessed the expression of these miR-449a in rat cortical neurons treated with neurotoxic Aβ_42_ peptide for 48 h, which is known to cause CRNA (9, 52, 53, 57). The expression of miR-449a was found to be significantly reduced in response to Aβ_42_ treatment (Fig. 3*A*) and was consistent with RNAseq analysis (Fig. 1, *A* and *B*), as well as reduced expression in TgAD neurons as mentioned above (Fig. 1*D*). These studies suggested that miR-449a is downregulated in response to aberrant Aβ_42_ production.

In addition, we were able to obtain frontal cortex tissue from two AD patients and age-matched normal individuals from a brain bank (details in Table S4). Interestingly, the expression of miR-449a was significantly lower in the cortex of two AD patients in comparison to healthy individuals (Fig. S1*C*), which supported studies performed in cultured neurons and raised the possibility of contribution of miR-449a in AD.

Given that miR-449a was downregulated in response to Aβ_42_ (Figs. 1*D* and 3*A*), it was relevant to investigate the outcome of its deregulation on cell cycle as it suppresses the neuronal cell cycle (Fig. 2). To address this, miR-449a was overexpressed using lentiviral transduction in rat cortical neurons followed by Aβ_42_ treatment or in neurons from TgAD mice. Previous studies have indicated that neurons undergo CRNA in these situations (52, 57). Western blotting revealed that PCNA, which was elevated due to Aβ_42_ treatment, was significantly downregulated upon miR-449a overexpression (Fig. 3*B*, lane 3 *versus* lane 2) along with reduced cleaved caspase 3 (Fig. 3*B*, lane 3 *versus* lane 2). Similar observations were also made when miR-449a (Fig. 3*C*) was overexpressed using lentiviral transduction in TgAD mouse cortical neurons. Phosphorylation of retinoblastoma protein is dependent on cyclin D expression as it activates CDK4/6 and is critical for S-phase entry (5). An increase in Rb phosphorylation was found in TgAD neurons, which was reduced upon miR-449a overexpression (Fig. S2*F*), which was consistent with reduced cell cycle entry indicated by PCNA expression and apoptosis by cleaved caspase 3.

BrdU incorporation and TUNEL labelling was performed as described above to detect the status of cell cycle and apoptosis, respectively. Aβ_42_ treatment resulted in a significant increase in TUNEL^+^/BrdU^+^ neurons consistent with previous studies and indicated that these neurons exhibited aberrant cell cycle reentry which results in apoptosis (CRNA) (52, 53, 57). A significant reduction in BrdU^+^/TUNEL^+^ cells was observed in Aβ_42_-treated cortical neurons when miR-449a was overexpressed (Fig. 3*D*). Collectively, these findings established that miR-449a plays an important role in preventing aberrant cell cycle reentry of neurons, which is induced by Aβ_42_ and results in apoptosis.

### miR-449a may prevent CRNA by targeting cyclin D1 and CDC25A

In order to decipher mechanisms *via* which miR-449a regulates the cell cycle during neuronal differentiation, the targets of this miRNA were predicted using *in silico* analysis. As mentioned above, three independent algorithms (miRDB, TargetScan, and RNA22) were used for this purpose (Fig. S2*A* and Dataset S4). Putative target sites for miR-449a were found in the 3′-UTR of several genes that have been associated with cell cycle progression, which include cyclin D1, CDC25A, cyclin E2, CDK6 (Fig. S2*A* and Dataset S4). It is important to note that these target sites are conserved in rodents and human (not shown here). Cyclin D1 is involved in G1 progression as it activates CDK4/6, and CDC25A is a phosphatase involved in cell cycle regulation as it activates CDKs by removing phosphates from their inhibitory phosphorylation site (T14, Y15 in CDK1) (74, 75). Previous studies have indicated that miR-449a may target cyclin D1 and CDC25A in dividing cells (45, 48, 76, 77, 78). Given that both cyclin D1 and CDC25A are critical for G1-S transition, it was reasonable to probe if miR-449a prevents CRNA by targeting these cell cycle regulators.

**Figure 4:**
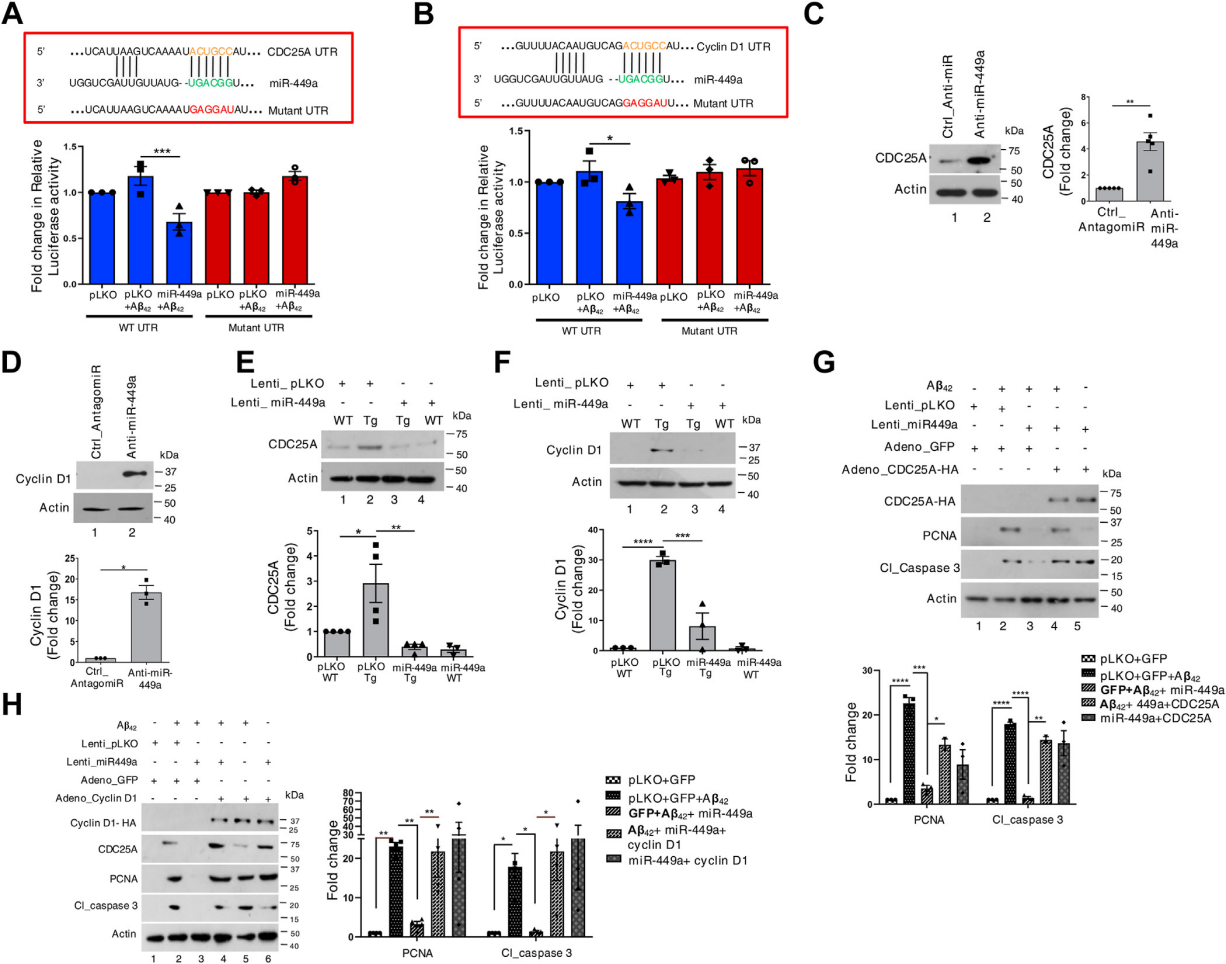
**Regulation of CDC25Aand Cyclin D1 by miR-449a may prevent CRNA.***A* and *B*, rat cortical neurons were transfected with a luciferase reporter plasmid containing CDC25A 3′-UTR (*A*) or cyclin D1 3′-UTR (*B*) along with transduction of miR-449a or pLKO (control) lentivirus and treated with Aβ_42_ for 48 h. In addition, a mutant in which miR-449a site in these UTRs was disrupted was also transfected. Cell lysates were prepared, luciferase activity assays were performed, and fold change in activity with respect to pLKO transduced cells was determined. Data represent mean ± SEM, two-tailed paired *t* test, ∗*p* < 0.05, ∗∗∗*p* < 0.001, N = 3. A predicted miR-449a–binding site in CDC25A (*A*) and cyclin D1- 3′-UTR (*B*) is indicated in *red*. *C* and *D*, rat cortical neurons were transfected with anti-miR-449a or control antagomiR. Neurons were harvested after 48 h and lysates were subjected to immunoblotting for CDC25A (*C*) or cyclin D1 (*D*). Actin was used as a loading control. Densitometry was performed to determine fold change in these proteins [*Right/bottom panel*, mean ± SEM, two tailed paired *t* test, ∗*p* < 0.05, ∗∗*p* < 0.01, N = 5 (*C*), N = 3 (*D*)]. *E* and *F*, cortical neurons from WT/TgAD mice were transduced with miR-449a or control lentivirus. After 48 h, lysates were prepared and Western blotting was performed with indicated antibodies. *Bottom panel*, densitometry was performed and fold change in cyclin D1 or CDC25A with respect to pLKO transduced WT neurons is provided (mean ± SEM, one-way ANOVA with Sidak multiple comparison, ∗*p* < 0.05, ∗∗*p* < 0.01, ∗∗∗*p* < 0.001, ∗∗∗∗*p* < 0.0001, N = 4 (*E*), N = 3 (*F*)). *G*, cortical neurons treated with Aβ_42_ for 48 h or left untreated were transduced with miR-449a or control lentivirus 48 h. Subsequently, neurons were also infected with adenovirus expressing CDC25A-HA or GFP (control). Western blotting was performed with indicated antibodies. *Bottom panel*, densitometry was performed to determine the levels of PCNA and cl_caspase 3 which were normalized with respect to actin and fold change in their expression with respect to pLKO-transduced neurons was determined. Data represent mean ± SEM, two-way ANOVA with Tukey’s multiple comparison, ∗*p* < 0.05, ∗∗*p* < 0.01, ∗∗∗*p* < 0.001, ∗∗∗∗*p* < 0.001, N = 3. *H*, rat cortical neurons were transduced with miR-449a or control lentivirus, treated with Aβ_42_ for 48 h or left untreated. Subsequently, neurons were also infected with adenovirus expressing cyclin D1-HA or GFP (control). Western blotting was performed with indicated antibodies. *Right panel*, densitometry was performed to determine the levels of PCNA and cl_caspase 3 which were normalized with respect to actin and fold change in their expression with respect to pLKO-transduced neurons was determined. Data represent mean ± SEM, two-way ANOVA with Tukey’s multiple comparison, ∗*p* < 0.05, ∗∗*p* < 0.01, N = 3.

To study if miR-449a targets 3′-UTR of cyclin D1 and CDC25A mRNAs, rat cortical neurons were transfected with luciferase reporter plasmid containing the 3′-UTR of cyclin D1 or CDC25A. Cotransfection of anti-miR-449a with cyclin D1 and CDC25A 3'-UTR in cortical neurons revealed a significant increase in luciferase activity, which indicated that miR-449a present in neurons can suppress cyclin D1 and CDC25A (Fig. S6). The overexpression of miR-449a suppressed luciferase activity in Aβ_42_-treated cells in both cases (Fig. 4, *A* and *B*). In contrast, luciferase activity for the mutant 3′-UTR in which the miR-449a target site was disrupted in both CDC25A and cyclin D1 remained almost unchanged. Importantly, transfection of anti-miR-449a in cortical neurons caused a significant increase in cyclin D1 and CDC25A mRNA (Fig. S2*B*) as well as protein (Fig. 4, *C* and *D*). Furthermore, the overexpression of miR-449a caused a significant decrease in both cyclin D1 and CDC25A, which exhibited an increase upon Aβ_42_ treatment (Fig. S2, *C* and *D*) or were upregulated in TgAD neurons (Fig. 4, *E* and *F*). These data suggested that miR-449a may repress the expression of key cell cycle regulators cyclin D1 and CDC25A in neurons and its downregulation by Aβ_42_ may contribute to the increase in CDC25A and cyclin D1 expression.

Next, we tested if the ability of miR-449a to regulate CDC25A or cyclin D1 contributes to CRNA. To this end, CDC25A was overexpressed using adenovirus in Aβ_42_-treated (Fig. 4*G*) or TgAD (Fig. S2*E*) neurons. Since overexpressed CDC25A did not possess the 3′-UTR, its expression was expected not to be influenced by miR-449a. The expression of PCNA and cleaved caspase 3 was determined, which increased in Aβ_42_-treated neurons (Fig. 4*G*, lane 2) and miR-449a overexpression impaired the expression of these proteins (Fig. 4*G*, lane 3 *versus* lane 2). Strikingly, when CDC25A was overexpressed along with miR-449a, the expression of these proteins was again observed (Fig. 4*G*, lane 4 *versus* lane 3), which suggested that miR-449a–mediated targeting of CDC25A may prevent CRNA. Similar experiments were performed to establish if miR-449a targeting of cyclin D1 also contributes to CRNA. In these experiments, the reversal of CRNA was also observed significantly upon overexpression of cyclin D1 along with miR-449a in Aβ_42_-treated neurons (Fig. 4*H*, lane 3 *versus* lane 4). Collectively, these data indicated that the ability of miR-449a to repress at least CDC25A and cyclin D1 significantly prevents aberrant cell cycle re-entry and apoptosis of neurons by Aβ_42_.

### Lentivirus expressing miR-449a improves learning and memory defects in APP/PS1 (TgAD) mice

Given that miR-449a expression is impaired in TgAD mouse neurons resulting in CRNA, it was pertinent to see if elevation in its expression prevents cognitive defects exhibited by these animals. Since previous studies (79) and our preliminary observations suggested that these defects are most apparent from ∼6 months of age, miR-449a-lentivirus was injected in animals of this age. Stereotaxic injections were performed in the frontal cortex as follows: a. present studies have been performed on cortical neurons from TgAD mice and consistent with previous reports (52, 53, 57) these neurons exhibit CRNA; b. previous studies on various AD mouse models have reported aberrant cell cycle re-entry of neurons in the cortex (9, 16); c. the neuronal loss in AD has been previously reported in the hippocampus as well as the cortex (80, 81, 82) and may contribute to AD-related cognitive defects.

**Figure 5:**
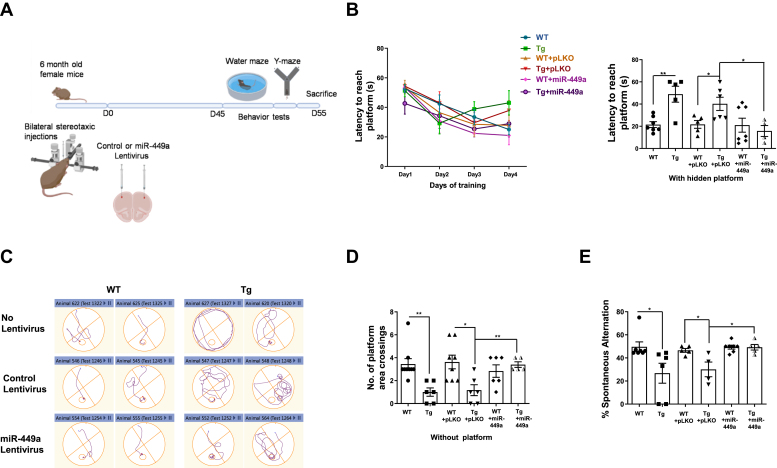
**miR-449a overexpression improves learning and memory in TgAD mice.***A*, schematic illustrating protocol followed for lentivirus injection and behavioral tests. miR-449a-LV or control pLKO-LV (without miRNA) were stereotaxically injected in the frontal cortex of 6M-old TgAD or WT female mice. Cognitive functions were accessed with the help of indicated behavioral tests that were conducted 45 days after injections. *B*, Morris Water Maze test was performed with a hidden probe to determine the mean escape latency to reach the platform at indicated time. *Right panel*, latency to reach the platform on the final day (Day 4) of training was found to be significantly reduced in miR-449a-LV–injected TgAD mice (Mean ± SEM, N = 5–8 mice/group; ∗*p* < 0.05; ∗∗*p* < 0.01, two-tailed unpaired *t* test). *C*, representative track plots of two mice from each group in MWM test described in Panel *B*. *D*, probe trial was performed 24 h after the last hidden platform test in the Morris water maze (*A*). The number of platform area crossings in the target quadrant is shown (mean ± SEM, N = 5–8 mice/group; ∗*p* < 0.05; ∗∗*p* < 0.01, two-tailed unpaired *t* test). *E*, for Y-maze test, mice were acclimatized in the maze with one arm closed for 3 min and then the alternations were recorded and % spontaneous alternation for each mouse was determined (See Experimental procedures). Data represent mean ± SEM, N = 5 to 8 mice/group, ∗*p* < 0.05, two-tailed unpaired *t* test.

A group of 6-month-old WT and TgAD female mice (split in six independent cohorts) (83) were injected bilaterally in the frontal cortex region with lentiviral particles encoding the miR-449a hairpin (miR-449a LV) (described above) or the negative control lentivirus (pLKO LV), which does not express any miRNA (Fig. S4). In addition, control experiments were also performed with animals that were not injected. Subsequently, memory and learning of these animals was assessed 45 days after injection (Fig. 5*A*).

Morris water maze (MWM) test was performed to evaluate the changes in spatial learning and memory of these animals (84, 85, 86) (Fig. 5*A*). An increase in latency in reaching the hidden platform was observed in the case of uninjected or control lentivirus (pLKO-LV)–injected TgAD mice relative to the corresponding WT groups, which was consistent with previous observations (87, 88, 89). Strikingly, the TgAD mice injected with miR-449a-LV displayed a significant decrease in the latency on the final day (day 4) of the trials (Fig. 5, *B* and *C*). It was important to assess the stored memory, which is regulated by the frontal cortex (90). Therefore, the probe trial of MWM test was performed in which TgAD mice exhibited decreased platform crossings than the WT animals as reported previously for AD mouse models (87, 88, 89). Upon miR-449a-LV injection, TgAD animals exhibited marked improvement in the target quadrant crossings compared to TgAD groups (uninjected or pLKO-LV injected) (Fig. 5*D*). There was no significant change in the average swimming speed of mice in each group (Fig. S3*B*). These data suggested that miR-449a-LV can improve spatial learning in TgAD mice.

The effect of the miR-449a on the working memory of TgAD mice was also evaluated by conducting the Y-maze task (Fig. 5*A*). The TgAD mice displayed a decrease in spontaneous alternation and no significant improvement was observed following control virus injections (pLKO-LV) in these animals (Fig. 5*E*). In comparison, TgAD-miR-449a-LV animal group showed a significant increase in the percentage of spontaneous alternations, which is an index of working memory (91, 92) (*p* < 0.05, Fig. 5*E*). There was no significant change in the number of arm entries among all the groups (Fig. S3*C*). Collectively, these studies strongly indicated that miR-449a lentiviral delivery improves learning and memory in TgAD mice.

## Discussion

After achieving terminal differentiation, the cell cycle of neurons remains suppressed for the remainder of their life span (1, 2). If the state of arrest is altered by insults like Aβ_42_, neurons attempt to re-enter the cell cycle, which predominantly results in their apoptosis (3, 14, 52, 57, 58, 93). It is still unclear how only a subset of apoptotic neurons die due to aberrant cell cycle re-entry. Nevertheless, they contribute to neuronal loss and neurodegeneration (14, 15, 93, 94, 95) in AD and other neurodegenerative disorders (96, 97, 98, 99, 100, 101).

We identified miRNA that exhibit altered levels of expression in the neurons of APP/PS1 (TgAD mice). On comparison with available data from published studies, we found that some of these miRNA have been previously reported to exhibit similar changes in the expression in *postmortem* brains/CSF of AD patients (60, 61, 62, 63, 64) and/or in studies directed at understanding neuronal apoptosis using primary cells or cell lines (30, 65, 66, 67, 68). Since several of these miRNA have putative target sites in the 3′-UTR of cell cycle–related genes, they are putative or established regulators of the cell cycle and are likely to regulate the neuronal cell cycle and possibly involved in CRNA. miR-449a is expressed both in embryonic (40, 41, 102, 103) and the adult cortex (104). Moreover, miR-34/449 KO mouse embryos exhibit a delay in neurogenesis, which has been attributed to its involvement in spindle misorientation in cortical progenitors (40). As a result, excess radial glial cells were formed at the expense of intermediate progenitors resulting in a significant delay in neurogenesis. miR-449a has been extensively studied in dividing cells and are negative regulators of cell division (45, 46, 47, 78, 105), which allows them to act as tumor suppressors (42, 105, 106, 107). It is apparent from these studies that miR-449a may act as a regulator of cell cycle in different settings. There is an interesting correlation between these and present studies, which clearly revealed that the ability of miR-449a to regulate neuronal cell cycle is critical for maintaining the differentiated state of cortical neurons as its depletion caused the neurons to make attempts to re-enter the cell cycle that leads to apoptosis instead of division (Fig. 2). These findings were also consistent with the role of miR-449a during differentiation of neuroblastoma cells induced by retinoic acid (47, 108). We found that the ability of miR-449a to promote neuronal differentiation by suppressing the cell cycle turns out to be critical for neuronal survival as its deregulation by Aβ_42_ causes dedifferentiation, resulting in aberrant cell cycle re-entry followed by apoptosis.

A decrease in the expression of miR-449a was observed in TgAD as well as Aβ_42_-treated neurons (Figs. 1*D* and 3*A*). Previous studies have also indicated that the expression of miR-449a in the CSF of AD patients may indeed be lower than normal individuals (109, 110). Detailed investigations revealed that miR-449a overexpression could revert the process of CRNA (Fig. 3, *B–D*).

Several putative targets of miR-449a were identified *in silico* that were related to the cell cycle and some of these were previously reported to be regulated by miR-449a (45, 77, 78, 107, 111). We focused on CDC25A and cyclin D1 as they are critical for G1-S transition (4, 112). Cyclin D1 interacts with CDK4/6 and results in kinase activation, which in turn phosphorylates Rb protein leading to E2F1 activation and downstream events necessary for S phase entry (112, 113). The activity of CDKs is regulated positively as well as negatively by phosphorylation. The inhibitory phosphorylation occurs at T14 and Y15 (a.a. numbers for CDK1) or corresponding sites (6). CDC25A is a phosphatase which dephosphorylates these sites to reactivate the G1 and S phase CDKs like CDK4/CDK6 (75, 114). Interestingly, miR-449a targets CDC25A and its expression is repressed in cancer cells and it is known to have tumor suppressor functions (35, 107). Aβ_42_ caused an increase in the expression of CDC25A and cyclin D1 (Fig. 4, *E* and *F* and Fig. S2, *C* and *D*), consistent with previous studies (15, 53, 74). While impaired miR-449a expression contributed to the increase in these cell cycle proteins, the fact that overexpression of CDC25A (Fig. 4*G* and Fig. S2*E*), and to some extent cyclin D1 (Fig. 4*H*), could revert CRNA suggested that miR-449a prevents CRNA by suppressing the expression of these proteins. Cyclin D1 has been shown to be upregulated in various AD models and contributes to CRNA (52, 53). CDC25A is upregulated in response to neurotoxic insults like DNA damaging agents and Aβ_42_ (74, 115, 116). Present studies provide a novel mechanism of its regulation by miR-449a *via* which it contributes to CRNA. Given that these cell cycle proteins are upregulated by Aβ_42_, it is reasonable to state that their repression by miR-449a may contribute to the prevention of CRNA.

**Figure 6:**
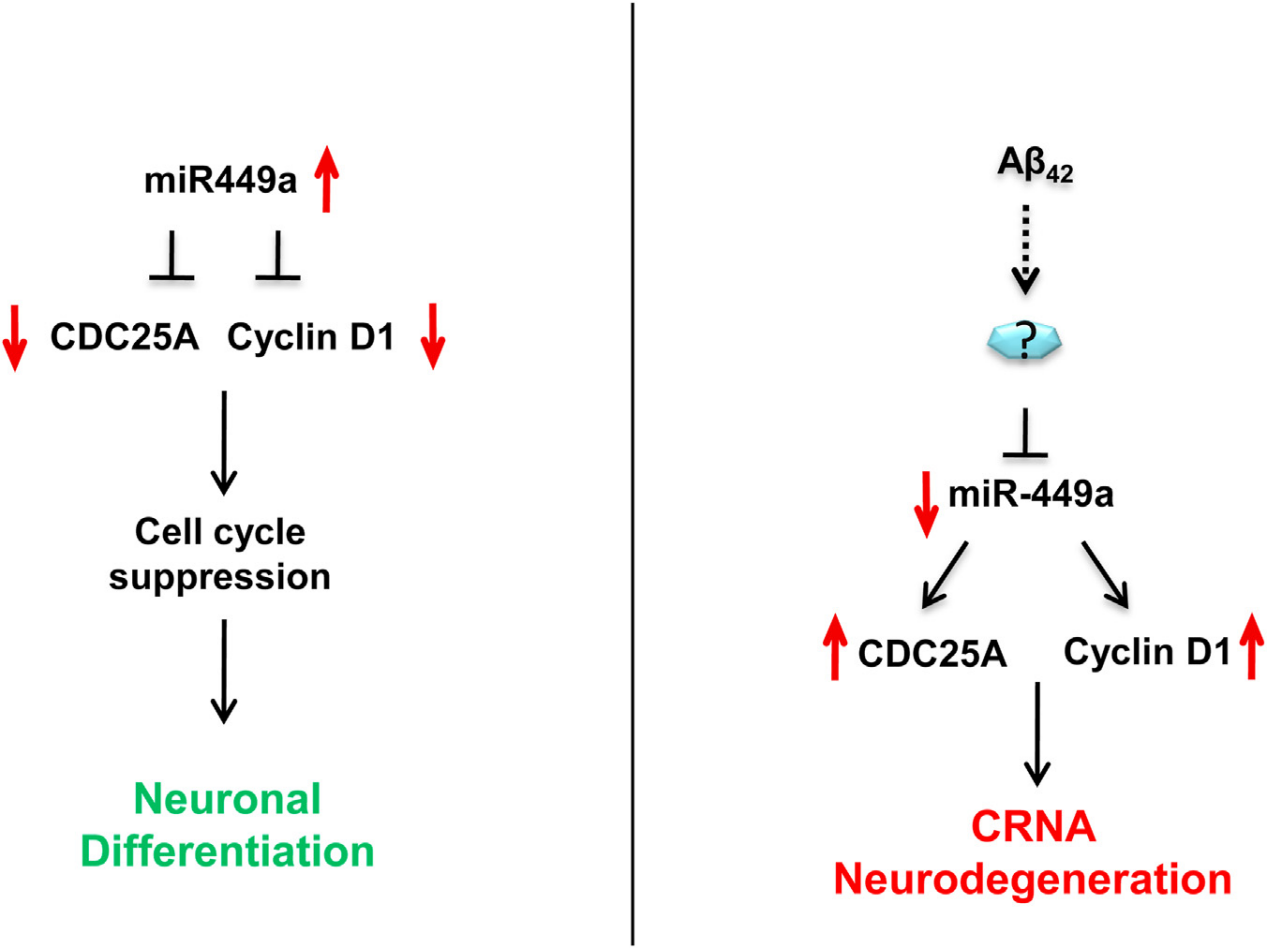
**Role of miR-449a in the regulation of neuronal cell cycle during differentiation and CRNA.** During neuronal differentiation, miR-449a is upregulated, which suppresses cell cycle regulators like cyclin D1 and CDC25A resulting in cell cycle arrest. Aβ_42_ impairs miR-449a expression via an unknown mechanism, resulting in an increase in CDC25A and cyclin D1, which promotes cell cycle re-entry resulting in the apoptosis of neurons. These events may contribute to cognitive defects associated with AD.

Various transgenic models for AD like APP/PS1 mice have been reported to exhibit cognitive defects reflected by impaired learning and memory (87, 88, 117), which is why they have served as a very useful model for studying AD pathology as well as developing anti-AD therapeutics (118, 119, 120, 121, 122). In the light of the *in vitro* results, we tested if miR-449a could effectively prevent learning and memory deficits in APP/PS1 TgAD mice. Since the focus of present studies was on cortical neurons in which CRNA has been observed in present and previous studies (9, 52, 53, 57), lentivirus were injected in the anterior region of the cortex. We found that lentivirus-mediated overexpression of miR-449a significantly reverted cognitive decline in TgAD mice, suggesting that it could be a good candidate for AD therapy (Fig. 5). The MWM test performed on these mice revealed a decreased learning ability of TgAD mice (Fig. 5*B*). The TgAD mice displayed no improvement in latency even after longer duration of training, which was consistent with previous reports (106, 123). Strikingly, miR-449a-LV–injected TgAD mice needed fewer days of training to match the performance of WT animals (Fig. 5, *B* and *C*), indicative of reversal in defects in learning. Strikingly, a marked improvement in long-term or retrograde memory—which was indicated in experiments performed without the platform—in miR-449a-LV–injected TgAD mice as these animals exhibited higher number of crossings in the platform area than the TgAD group (Fig. 5*D*). Spatial or working memory assessed by Y-maze test (91, 92) indicated that miR-449a-LV–injected TgAD mice showed a significant improvement compared to control mice (Fig. 5*E*). In the light of our present findings, it is reasonable to speculate that the reversal of cognitive defects by miR-449a in TgAD mice may be due to prevention of neuronal apoptosis caused by cell cycle re-entry. Present studies highlight a dual role of miR-449a in neurons: on one hand, miR-449a suppress the neuronal cell cycle to promote neuronal differentiation and maintain the state of differentiation and on the other, it prevents aberrant cell cycle re-entry of neurons, which results in neuronal cell death in response to Aβ_42_ (Fig. 6). Present studies also highlight therapeutic potential of miR-449a in providing relief to AD patients by improving cognitive defects *via* the mechanism reported in the present work.

## Experimental procedures

Information related to antibodies and other reagents is provided in Supporting information file.

Mice and rats used in this study were housed in standard pathogen free conditions in National Institute of Immunology animal housing, maintained on a 12 h light and dark cycle with food and water access to *ad libitum*. All animal handling procedures were followed as per the guidelines established by Institutional Animal Ethics Committee (IAEC) and all the experiments were conducted in compliance with NIH guidelines.

### APP/PS1 (TgAD) Alzheimer’s disease mouse model

Amyloid Precursor Protein/Presenilin 1 (APP/PS1) transgenic mouse model for AD (TgAD) (strain B6C3-Tg APPswe, PSEN1dE9 85Dbo/J; stock number 004462-JAX) maintained at the Jackson laboratory was a kind gift from National Brain Research Centre, Manesar to National Institute of Immunology (NII), New Delhi. The APP/PS1 (TgAD) double transgenic mice express a chimeric mouse/human amyloid-β precursor protein containing K595N and M596L Swedish mutations, a mutant human presenilin1 gene carrying the exon-9 deletion under the control of mouse prion promoter elements that direct transgene expression predominantly in the central nervous system neurons. The levels of Aβ_42_ produced are significantly higher in female animals (54, 55). WT and transgenic (TgAD) mice were genotyped using genomic DNA isolated from mouse tail and PCR primers as suggested by Jackson’s laboratory. After genotyping, mice were randomly divided into experimental groups (n = 8 per group). Typically, five or less mice were housed together in one cage.

### Cell culture

Cortical neurons from embryonic day 18 (E18) Sprague-Dawley rats or embryonic day 16 (E16) APP/PS1 transgenic AD mice were isolated and cultured as previously published (52, 53). Six-to-eight-week old male and female mice/rats were put together in a 2:1 harem and the males were separated after 2 days. The females were checked for pregnancy after 10 days. Only two mated females were housed together and always handled gently to avoid any trauma. Between 16-18 day, the pregnant rat/mouse was euthanized with CO_2_ overdose and the embryos were kept on ice for 10 min for euthanasia following IAEC guidelines. Subsequently, E18 rat or E16 mouse embryos were dissected and cortical region of the brain was isolated and treated with Trypsin (cat. no.:T4799 from Merck)-DNase (cat. no.: DD0099 from Link Biotech) followed by the addition of serum-containing media and centrifugation at 500*g* for 5 min at room temperature. Cell pellet was resuspended in serum-containing media and plated on poly-L-Lysine (cat. no.: P2636 from Merck) coated 6-well (35 mm) plates. After 12 h, cells were washed with Tyrode’s CMF PBS supplemented with glucose and NaHCO_3_ and were maintained in serum-free medium containing B27 and N2 supplement (cat. no.: 17504044 and 17502048, respectively from Gibco, Life technologies), 1× penicillin–streptomycin (cat. no.: 15140148, all from Gibco, Life technologies), L-glutamine (cat. no.: G8540 from Merck), and glucose maintained at 5% CO_2_ levels. Typically, *in vitro* transfections or Aβ_1-42_ treatments were performed at DIV5.

HEK293T/A (Human Embryonic Kidney) cells (American Type Culture Collection) were maintained in Dulbecco’s modified Eagle’s medium (cat. no.: 12800017) with 10% (vol/vol) fetal bovine serum (cat. no.: 10270106) and 1× antibiotic/antimycotic (cat. no.: 15240-062, all from Thermo Fisher scientific) at 37 °C at 5% CO_2_. Cells within 15 passages after purchase were used in this study. All experiments were performed in accordance with the guidelines for animal experiments of the IAEC, National Institute of Immunology.

One pregnant rat yielded ∼ 15 to 20 embryos; brains from these embryos were further pooled to obtain primary cortical neurons (∼50 × 10^6^). These isolated neurons were cultured and subjected to various treatments as per the need of the experiment and this was considered as an independent biological replicate. In case of the TgAD mice, cortical neurons from each individual embryo were cultured separately. Subsequently, genotyping was done to identify the WT and TgAD neurons. These neurons were subjected to desired treatments and were considered as a biological replicate and at least three biological replicates were performed for each experiment.

### RNA isolation and qRT-PCR

After the desired treatments, total RNA was isolated by using TRIzol reagent (cat. no.: 10296010 from Thermo Fisher Scientific). Briefly, samples in TRIzol reagent were homogenized and kept at room temperature for 5 min, followed by a chloroform (cat. no.: 1.94506) wash. 3M sodium acetate (cat. no.: S2889) (pH 5.2) and 1 μl of 50 mg/ml glycogen (cat. no.: 361507) (for small RNA isolation only) and isopropanol (cat. no.: 34863) were added for precipitating RNA, which was washed twice with 75% ethanol (cat no.: 1.07017, all from Merck), followed by resuspension in nuclease-free water.

qRT-PCR for microRNA was performed by using a TaqMan microRNA assay kit. Typically, 1 μg of total RNA was reverse transcribed using a TaqMan microRNA RT kit (Applied Biosystems) and microRNA-specific stem-loop RT primers provided in the TaqMan microRNA assay kit (miR-449a-001030, RNU6B-001093). qRT-PCR was performed in a CFX96 real time thermo cycler (Bio-Rad). The expression of miRNA was defined on the basis of threshold cycle (C_T_), and the relative expression levels were determined by ΔΔC_T_ after normalization with RNU6B.

### RNA sequencing and data analysis

Small RNA sequencing was performed on two biological replicates and was outsourced to Novogene by Bencos Research Solutions and was performed on an Illumina HiSeq 2500 platform (50 bp single-end reads). Compressed (.gz) clean sequencing files (adapters removed) in fastq format were obtained from the service provider. Reads were mapped to the mouse genome GRCm38 version using Bowtie2 (124) without mismatch. HTseq-count was used to count the number of reads per microRNA gene with a gff3 annotation file from miRBase release 22.1. Differential expression analysis of miRNA in WT and TgAD neuron samples with two biological replicates was performed using the DESeq2 (125). P-adjusted and log2 (fold change) cutoffs used to distinguish differentially expressed genes are described in the text.

### Transfection and treatments

Lipofectamine 2000 reagent (cat. no.: 11668027 from Invitrogen) was used for the transfection of plasmid DNA and antagomir according to manufacturer’s instructions. Cortical neurons were transfected with 1-3 μg of plasmid DNA or 100 pmoles of anti-miRNA (antagomir) per well in a six well plate in serum-free medium without antibiotic. After 3 to 4 h of transfection, cultures were moved to medium with supplements and antibiotic. Typically, 0.5 μM of soluble oligomers of Aβ_1-42_ (cat. no.: A-1163 from R-peptide) was used for the treatment of cortical neurons for 48 h, to induce CRNA as described previously (52, 53, 126).

### Viral production and transductions

Two types of viral gene delivery systems were used in this study. Lentivirus system was used for miRNA expression (127) and adenovirus system for gene expression (53) in post-mitotic neurons.

#### Lentivirus

Oligonucleotides containing miRNA hairpin loop sequence (obtained from miRDB) or unrelated sequence (negative control) with *EcoR*I and *Pac*I restriction site overhangs were commercially synthesized from Merck. Oligos were annealed and cloned in pLKO.3G (cat. no.: 14748) vector and this plasmid construct along with packaging plasmids pCMV-vsv-g and pΔR8.2-dvpr (cat. no.: 8454 and 8455 from Addgene) were cotransfected in HEK293T cells (128, 129). Supernatant was collected 40 h and 72 h post transfection, filtered, and used to estimate the number of transducing units (TU/ml) by GFP fluorescence after 24 to 48 h and was used to transduce cultured primary cortical neurons. Lenti-X concentrator solution (cat. no.: 631231 from Takara Bio Inc.) was used for concentrating the virus to be injected in the brains at a concentration at ∼10^6^ to 10^7^ TU/ml.

#### Adenovirus

Adenovirus constructs were prepared for CDC25A and cyclin D1 (details in Supplementary methods) expression in post mitotic neurons. pAdTrack (cat. no.: 16404) shuttle vector constructs were generated by cloning relevant cDNA, and the resultant plasmids were digested with *PmeI* followed by electroporation in *Escherichia coli* BJ5138 containing pAdEasy-1 (cat. no.: 16400, both from Addgene) vector. The recombinant clones were digested with *Pac* I and transfected in HEK293A cells. The adenovirus was harvested and amplified using standard procedures (130, 131, 132). Adenovirus, at ∼10 multiplicity of infection, was incubated with cortical neurons and efficiency of infection was determined by observing GFP fluorescence after 24-48 h.

### Immunoblotting

Cells were washed with 1× PBS and lysed using ice cold lysis buffer (100 mM Tris–HCl pH 7.4, 5 mM EDTA, 100 mM NaCl, 1% Triton ×100 and 10% Glycerol, 1 mM PMSF, 1 mM sodium orthovanadate, 20 mM β-glycero-phosphate, and 1× protease inhibitor cocktail was added before use). Immunoblotting was performed after transferring proteins to nitrocellulose membrane as described previously (52) using primary antibodies and secondary antibody conjugated with horse radish peroxidase. Subsequently, membranes were incubated with the chemiluminescence reagent West Pico or West Dura (Pierce) (Cat. No.: 32209 from Thermo Fisher scientific), which was used for detection as per manufacturer's instructions by exposing the blots to X-ray film.

### BrdU incorporation and TUNEL assay

5-bromo-2′-deoxyuridine (BrdU) labelling was performed to detect DNA replication. Ten micromolars of BrdU was added in the media of primary cortical neurons; a fresh pulse of BrdU was given every 4-6 h for 48 h. Anti-BrdU antibody (1:50, cat. no.: RPN202, from GE Healthcare Bio-Sciences) was used to detect incorporated BrdU (52, 53). Terminal deoxynucleotidyl transferase dUTP nick end labeling (TUNEL) assay to detect cell death was performed by using Dead End fluorometric TUNEL system (cat. no.: G3250, Promega) as per manufacturer’s guidelines and Hoechst 33342 (cat. no.: 33342, Molecular Probes) was used to stain the nuclei. These two assays were performed simultaneously (52, 57) and labeled cells were visualized using a Zeiss AxioImager1 microscope, and Axiovision software was used for image acquisition and processing images and population of cells positive for BrdU and/or TUNEL was determined.

### Luciferase reporter assays

Cyclin D1/CDC25A 3′-UTR was amplified using primers mentioned in Table S2 using rat cDNA template and cloned downstream (details in Supplementary methods) of the Renilla luciferase gene in psiCHECK2 (cat. no.: C8021 from Promega) plasmid containing synthetic firefly luciferase gene (transfection control). The miRNA-binding sites in 3′-UTR were mutated for each UTR construct (see details in Supporting information). Transfections of these plasmid constructs in rat cortical neurons were performed using Lipofectamine 2000 and cells were harvested after 48 h and frozen at −80 °C for at least 24 h. After brief centrifugation, luciferase and renilla activity were measured with a dual-luciferase reporter assay kit (cat. no.: E1910, Promega).

### Lentivirus stereotaxic injections

WT/TgAD 6-month-old female mice (weighing 18–21 g) were anaesthetized with a ketamine/xylazine solution (75 mg/kg ketamine + 10 mg/kg xylazine in saline, intramuscular injection). The effect of anesthesia was verified every 3 min by toe pinching throughout the procedure. Lentivirus-encoding miR-449a precursor sequence or a control lentivirus (pLKO) lacking miRNA sequence (∼10^6^–10^7^ TU/ml) in a final volume of 3 μl were stereotaxically injected in both hemispheres of the cortex at the following coordinates as anterior/posterior: +1.8 mm; mediolateral: ± 1.9 mm; dorsal/ventral: −1.2 mm to the Bregma (133). The viral suspensions were injected at the flow rate 1 μl/min (Stoelting) and the needle-wound was closed and skin was sutured. As a part of post-operative care, mice were checked every day for any signs of swelling or infection and provided moist food for 3 days. Neosporin was applied on the wounds if and when required. Animals were monitored during recovery and subject to behavioral tests after 45 days. After completion of the behavioral tests, the animals were euthanized as per ethical guidelines.

### MWM test

After 45 days, all groups of mice indicated above were subjected to MWM test to examine memory and learning capacities. The test was performed for 4 days with the platform followed by a probe trial (without platform). Each mouse received three trials (1 min each) per day for four consecutive days (84, 85). For each trial, mice were placed in the pool at a different start location and were allowed to swim until they either located the hidden platform or reached the end of the 60s trial. Following completion of 4 days of testing, mice were subjected to a probe trial in which the platform was removed and swimming behavior was monitored for 60s. The learning of the platform location was evaluated by escape latency (the duration to reach the platform) during the training trials, and behavior during the probe trial was measured as time spent in the platform quadrant. All events were monitored and observed in person as well as with the help of camera recordings in ANY-maze software (Stoelting Co). Data from the animals were collected based on the mouse number and blinding was done during data analysis.

### Y-maze test

Short term memory was evaluated with the help of Y-maze task as described previously (134). Briefly, mice were randomly placed in one of the arms of the maze with one arm blocked and allowed to move for 3 min, and subsequently, block to arm was removed and the animal was allowed to move freely for another 5 min. The series of arm entries were recorded visually and with camera setup connected of ANY-maze software. Spontaneous alternations as short term memory index were calculated using the following equation:

$$\%\;\text{spontaneous}\;\text{alternation}\;\left(\text{SA}\right)=\frac{\text{No}.\;\text{of}\;\text{alternations}\;}{\text{No}.\;\text{of}\;\text{arm}\;\text{entries}-2}*100$$

### Image and statistical analysis

Image J (NIH) software was used for densitometry analysis of desired bands in Western blots. The band intensity of the loading control (β-Actin) was used for the normalization. Data are represented as mean ± SEM from three biological replicates (N), unless indicated otherwise. For the comparison between two groups, we used two-tailed *t* test and for several groups, ANOVA, followed by *post hoc* tests (Graph Pad Prism software Inc). Significant results were analyzed by *post hoc* Tukey’s test unless indicated otherwise. Differences between two groups were considered significant when *p* < 0.05.

### Ethical clearances

All the experiments were designed and performed in accordance with the guidelines of Institutional Ethics Committee. Animal experiments were approved under protocol #461/18 and #513/19 by Institutional Animal Ethics Committee. Human brain samples acquisition and usage was approved under protocol #120/19 by the Institutional Human Ethics Committee. This study was performed according to ethics committee approval and is compliant with "Declaration of Helsinki". All experiments involving use of lenti- or adenovirus were approved by Institutional Bio Safety committee under IBSC #346/19.

## Data availability

All the raw sequencing data generated in this study have been submitted to the NCBI GEO (https://www.ncbi.nlm.nih.gov/geo/) and can be accessed by the reviewers using the following link and token.

GEO accession GSE201209: https://www.ncbi.nlm.nih.gov/geo/query/acc.cgi?acc=GSE201209.

Token: ujqvmakudbudnmh.

## Supporting information

This article contains supporting information (135, 136, 137, 138, 139).

## Conflicts of interest

The authors declare that there is no conflicts of interest with the contents of this article.
