# Supplementary material for: miR-449a mediated repression of the cell cycle machinery prevents neuronal apoptosis[image]
Source: J Biol Chem. 2024 Aug 22;300(9):107698. doi: 10.1016/j.jbc.2024.107698 (PMC11419829; doi:10.1016/j.jbc.2024.107698)
Supplement: Supporting Information [file mmc1.pdf]

Supporting Information

Materials and Methods

1. Antibodies: Antibodies were used against the following proteins: PCNA (sc-56, 1:500 for IB), β-actin (sc-47778, 1:2000), HA probe F-7 (sc-7392, 1:500), were from Santa Cruz Biotechnology, Santa Cruz, CA. Antibody against cleaved caspase 3 (D-175) (#9661, 1:500), cyclin D1 (92G2) (#2978, 1:500) were from Cell Signaling technology. Anti-CDC25A (MA5-13794, 1:1000) antibody was from Invitrogen. Anti-GFP (11814460001, 1:100 for IHC) from Roche Diagnostics and anti-BrdU (RPN202, 1:50 for IFA) from GE Healthcare Bio-Sciences, Piscataway, NJ.

2. AntagomiR for miR-449a: miRNA inhibitor negative control #1 (4464076), miR-449a inhibitor (AM17000) were purchased from Ambion (USA). Each miRNA inhibitor was transfected with Lipofectamine 2000 at a concentration of 100 pico moles per well of a 6 well plate.

3. Plasmid DNA constructs

a. *psiCHECK-CDC25A3'-UTR*. A 492-bp fragment of the human CDC25A 3'-untranslated region (UTR) containing the putative miR-449a binding site was PCR amplified from HEK293 human cell line and cloned into the *Xho*I and *Not*I sites of the psiCHECK2 vector upstream of the Renilla luciferase gene using CDC25 WT_UTR1 and CDC25WT_UTR2 primer pairs mentioned in Supp. Table S2. The mutant of the miR-449a binding site at 516-522 bp was generated by PCR, which was performed using CDC25mutUTR1 and CDC25mutUTR2 primers (Supp. Table S2). All the positive clones were confirmed by sequencing. It is important to note

that miR-449a binding site in CDC25a-3'UTR is conserved in rat, mice and human (not shown here).

b. *psiCHECK-cyclin D1 3'-UTR.* Since this construct has been described previously and was available in the laboratory (1), it was used for present studies. The mutant of the miR-449a binding site at 2076-2082 bp was generated by mutagenesis PCR, which was performed using D1mutUTR1 and D1mutUTR2 primers (Supp. Table S2). All the positive clones were confirmed by sequencing (1). It is important to note that miR-449a binding site in cyclin D1-3'UTR is conserved in rat, mice and human (not shown here).

c. *pAdTrack-CDC25A.* For overexpression of CDC25A in primary cortical neurons, mouse CDC25A protein coding sequence along with C-terminus HA-tag was cloned in pAdTrack shuttle vector. Briefly, coding sequence for *CDC25A* was PCR amplified from mouse cDNA with CDC25A_adeno1 and CDC25A_adeno2 primers and cloned using the *Not*I and *Hind*III restriction sites of pAdTrack-CMV vector. CDC25A-HA-pAdTrack shuttle vector construct was digested with *Pme*I, and electroporated in *Escherichia coli* BJ5138 containing pAdEasy-1 vector. The recombinant clones were digested with *Pac*I before transfection in HEK293A cells.

d. *pAdTrack-Cyclin D1*. For the overexpression of cyclin D1 in primary cortical neurons, cyclin D1 adenovirus was used which was previously prepared in the laboratory (2).

5. Thioflavin Staining: The cryopreserved brain sections (10 µm thick) were thawed at room temperature followed by dehydration in 70% and 80% ethanol for one minute each. The slides were then dipped into the filtered solution of Thioflavin T (0.002% in 80% ethanol) for 8 min.

Slides were then washed in decreasing concentrations of ethanol from 80%, 70%, 50%, 50% and finally in distilled water for 2 min, air dried and mounted in DAPI containing mounting media (3,4). Brain sections encompassing the cortex region were stained for Thioflavin T (ThT). These ThT-stained brain sections were imaged with a 20x objective using a Zeiss microscope (Zeiss, Germany) for quantification. The intensity of ThT staining was quantified using Image J (NIH) (5).

6. Human Tissue lysate preparation for total RNA isolation: Frontal cortex tissue from two AD patients and age-matched normal individuals were acquired from National Institute of Mental Health and Neurosciences brain bank to assess the level of these miRNA. Human brain samples acquisition and usage was approved under the Institutional Human Ethics Committee with IHEC serial #120/19. The human studies reported on this manuscript abide by the Declaration of Helsinki principles. There was no requirement of the patient consents since these tissues were acquired from a tissue bank. For RNA isolation, 20-25 mg of tissue was homogenised in Trizol reagent (cat. no.: 10296010 from Thermo Fisher Scientific, USA) and proceeded as in main text.

Supplementary Figure legends

Supp. Figure S1

A. A flow chart showing the miRNAseq analysis pipeline and the tools used for the analysis.

B. A Venn diagram showing miRNA that were significantly altered in TgAD neurons (fold change cut off < 0.6 and padj cutoff< 0.1 in the present study and were also previously reported to be aberrantly expressed in human AD brain and AD mouse brain/AD cell culture models. A list of miRNA that are common in all three datasets are listed on the right.

C. RNA was isolated from the frontal cortex tissue of two AD patients or age matched healthy controls. miRNA expression was determined using qRT-PCR. Fold change in the expression of miRNA with respect to healthy individual is provided from three technical replicates for each of the two brains (mean ± SEM, n=3 of two biological samples, *p<0.05, **p<0.01, two tailed paired t-test, N=2).

Supp. Figure S2

A. miR-449a target prediction was done using indicated algorithms and databases. Venn diagram showing seven common targets predicted with miRDB, TargetScan and RNA22, which are indicated in the list (Supplementary Dataset S4).

B. Rat cortical neurons were transfected with anti-miR-449a or a control inhibitor. Total RNA was isolated and qRT-PCR was performed to determine the levels of CDC25A or cyclin D1 and fold change expression with respect to control inhibitor transfected neurons was determined (mean ± SEM, two tailed paired t-test, *p<0.05, N=3).

C. Rat cortical neurons were transduced with miR-449a or control lentivirus, treated with $A\beta_{42}$. After 48h, samples were harvested for RNA and protein analysis. Western blotting was performed with indicated antibodies. CDC25A levels were normalized with respect to actin.

Lower panel, Densitometry was performed on Western blots and fold change with respect to pLKO transduced cells is provided (mean±SEM, one-way ANOVA with Sidak's multiple comparison, **p<0.01, ***p<0.001, N=3).

Right panel, RNA was isolated and qRT-PCR was performed to determine the levels of CDC25A and fold change expression with respect to control pLKO transduced neurons was determined (mean ± SEM, one-way ANOVA with Sidak's multiple comparison, *p<0.05, N=4).

D. Rat cortical neurons were transduced with miR-449a or control lentivirus, treated or left untreated with $A\beta_{42}$. After 48h, samples were harvested for RNA and protein analysis. Western blotting was performed with indicated antibodies. Cyclin D1 levels were normalized with respect to actin.

Lower panel, Densitometry was performed on Western blots and fold change with respect to pLKO transduced cells is provided (mean±SEM, one-way ANOVA with Sidak's multiple comparison, ***p<0.001, ****p<0.0001 N=3).

Right panel, RNA was isolated and qRT-PCR was performed to determine the levels of cyclin D1 and fold change expression with respect to control pLKO transduced neurons was determined (mean±SEM, one-way ANOVA with Sidak's multiple comparison, ***p<0.001, N=5).

E. Cortical neurons cultured from WT or TgAD mice were transduced with miR-449a or control lentivirus for 48h. Subsequently, neurons were also infected with adenovirus expressing CDC25A or GFP (control). Western blotting was performed with indicated antibodies. *Right Panel*, Densitometry was performed to determine the level of PCNA and cl_caspase 3, which was normalized with respect to actin and fold change in expression with respect to pLKO transduced neurons was determined. Data represent mean±SEM of at least three replicates, *p<0.05, **p<0.01 with two tailed paired t-test, N=3.

F. Cortical neurons from WT and TgAD mice were transduced with miR-449a or control lentivirus. The lysate was prepared and Western blotting was performed to determine the phosphorylated Retinoblastoma (pRb) and total Rb proteins.

Supp. Figure S3

A. Rat cortical neurons were transfected with anti-miR-449a or a control inhibitor. RNA was isolated and qRT-PCR was performed for miR-449a. Fold change in expression with respect to control inhibitor transfected neurons was determined (mean ± SEM, * p<0.05 by two tailed paired t-test, N=2).

B. Average swimming speed (m/s) of mice from each group in Morris water maze probe test (Fig. 5D) is provided. No significant change in the average swimming speed was observed amongst the lentivirus (control or miR-449a) injected groups. Data represented as mean±SEM, N= 5-8 animals from each cohort, ns-not significant by one-way ANOVA with Tukey's multiple comparison.

C. Number of total arm entries in Y maze (Fig. 5E) is shown. No significant change was observed in the total number of arm entries of mice in each group. Data represented as mean ± SEM, N=6-8 animals from each cohort, not significant by one-way ANOVA with Tukey's multiple comparison.

Supp. Figure S4

Immunohistochemistry was performed on the brain sections of lentivirus-injected mice for the expression of GFP. Representative overview images showing GFP expression in the right hemisphere of the brain of TgAD mice injected with pLKO or miR-449a lentivirus in the cortex, image of age matched WT brain without any intervention is also provided (scale bar 100 μm). The images were stitched in the original settings in Zeiss microscope software.

Supp. Figure S5

A. Thioflavin T-stained brain sections encompassing the cortex of miR-449a or control (pLKO) lentivirus injected TgAD and age-matched WT and TgAD mice (scale bar: 100μm, 20x magnification) along with total cells in the field (DAPI stained).

B. Quantification of intensity of Thioflavin T-staining in plaques in coronal brain, which were measured from random sections from experiment described in panel A. Data is provided for WT and TgAD mice injected with with miR-449a or control (pLKO) mice (Mean ± SEM of N=3 animals per group and n=4 sections per animal, ns-not significant, ***p<0.0001 by two-way ANOVA with Sidak's multiple comparison) (5).

Supp. Figure S6

Cortical neurons from rat were cultured and transfected with luciferase reporter plasmid containing CDC25A 3′-UTR (A) or cyclin D1 3′-UTR (B), a control plasmid pRL-TK followed by transfection of a control antagomiR or anti-miR-449a. The lysates were prepared after 48h, luciferase activity assays were performed, and fold change in luciferase intensity with respect to control antagomiR transfected cells was determined. Data represent mean±SEM, two-way ANOVA with Sidak's multiple comparison, ****p<0.0001, ns not significant, N=3.

Supplementary Tables

Table S1. Predicted cell cycle related targets of candidate miRNA. *In silico* analysis was done using tools like Target Scan, miRDB, and RNA22 to predict putative targets of candidate miRNA that were aberrantly expressed in TgAD neurons. Some of the target genes that may be involved in cell cycle regulation are indicated.

Differentially expressed miRNA	Putative targets related to Cell cycle regulation
**miR-449a-5p**	CDK6, CDC5A, MEK1, CCND1, CCNE2
**miR-16-5p**	CCND1, CCNE1, CDK4
**miR-101c**	-
**miR-190a-5p**	CCND2
**miR-149-5p**	CDK17
**miR-29c-3p**	CCNA2, CCND2, CDK6, E2F2
**miR-128-3p**	CCNK, CCNC, CDK18
**miR-34c-5p**	CCND1, CCNE2, MEK1, CCNA1, CDK6
**miR-369-3p**	CCNE2, Rb1
**miR-30b-5p**	CCNA1, CCNE1, CCNE2
**miR-744-5p**	CCNA1, CCNE2, CCND1, CDK2, CDK4

**miR-3068-3p**	CDK6

Table S2. Details of PCR primers used for cloning:

S.No.	Primer	Sequence	Remarks
**1**	miR449a oligo1	aattcCTGTGTGTGATGAGCTGGCAGTGTATTGTTAGCTGGTTGAATATGTGAATGGCATCGGCTAACATGCAACTGCTGTCTTATTGCATATACAttaat	Cloning in pLKO.3G (Forward)
**2**	miR449a oligo 2	taaTGTATATGCAATAAGACAGCAGTTGCATGTTAGCCGATGCCATTCACATATTCAACCAGCTAACAATACACTGCCAGCTCATCACACAGg	Cloning in pLKO.3G (Reverse)
**3**	cdc25WT UTR1	CCCctcgagGAGGGGAGTAGAGAAGTTAC	Cloning in psiCHECK2 (Forward)
**4**	cdc25WT UTR2	CCgcggccgcCACCTCCCACCAAATAGATA	Cloning in psiCHECK2 (Reverse)
**5**	cdc25mut UTR1	GGTTGAAATGTGGACCTTCTAGGTA	SDM in psiCHECK2 (Forward)
**6**	cdc25mut UTR2	TACCTAGAAGGTCCACATTTCAACC	SDM in psiCHECK2 (Reverse)
**7**	D1mutUTR1	CAATGTCATAAGTACCTCTGTTCAAGTTTTAATTTCCTCGTAG	SDM in psiCHECK2 (Forward)
**8**	D1mutUTR2	CTACGAGGAAATTAAAACTTGAACAGAGGTACTTATGACATTG	SDM in psiCHECK2 (Reverse)
**9**	CDC25A_adeno1	TTgcggccgcATGGAACTGGGCCCGAGCCC	Cloning in pAdTrack-1 (Forward)
**10**	CDC25A_adeno2	CAAGCTTTCAAGCGTAATCTGGAACATCGTATGGGTAGAGCTTCTTCAGGCGAC	Cloning in pAdTrack-1 (Reverse) HA tag

Table S3. Primers used for real time PCR:

S.No.	Primer	Sequence
**1**	CDC25A_F	TAAGACCTGTATCTCGTGGCTG
**2**	CDC25A_R	CCCTGGTTCACTGCTATCTCT
**3**	cyclinD1_F	CGCCTTCCGTTTCTTACTTCA
**4**	cyclinD1_R	AACTTCTCGGCAGTCAGGGGA
**5**	GAPDH_F	GGTCGGTGTGAACGGATTTG
**6**	GAPDH_R	CATGTAGACCATGTAGTTGAGGT

Table S4. Details of Human brain tissue from NIMHANS Brain Bank:

Human brain tissue repository (HBTR) No.	Diagnosis/ Anatomical area	Age/Sex	PMI
12/B243	Alzheimer's/ Frontal	82/ F	--
15/B271	Alzheimer's/ Frontal	92/ F	4h 36min
16/B281	Normal Control/ Frontal	95/ F	11h
16/B282	Normal Control/ Frontal	80/ F	16h 45min

Table S5. Details of exact P value for respective main figure comparisons.

Figure	Comparison	p-value
**1D**	**miR-449a**	
	WT vs. TgAD	0.0001
**2A**	**miR-449a**	

	DIV2 vs. DIV5	0.438
	DIV2 vs. DIV7	0.0086
	DIV5 vs. DIV7	0.0643
**2B**	**PCNA**	
	Ctrl vs. anti-449a	0.0073
	**Cl_caspase3**	
	Ctrl vs. anti-449a	0.0156
**2C**	**TUNEL+BrDU+**	
	Ctrl vs. anti-449a	0.0101
**3A**	**miR-449a**	
	Ctrl vs. Aβ	0.0052
**3B**	**PCNA**	
	pLKO vs. pLKO+ $A\beta_{42}$	0.0157
	pLKO+ $A\beta_{42}$ vs. miR-449a+ $A\beta_{42}$	0.0420
	**Cl Caspase3**	
	pLKO vs. pLKO+ $A\beta_{42}$	0.0006
	pLKO+ $A\beta_{42}$ vs. miR-449a+ $A\beta_{42}$	0.0115
**3C**	**PCNA**	
	WT pLKO vs. Tg pLKO	0.0005
	Tg pLKO vs. Tg miR-449a	0.0017
	**Cl Caspase3**	
	WT pLKO vs. Tg pLKO	0.0001
	Tg pLKO vs. Tg miR-449a	0.0001

**3D**	**TUNEL+BrDU+**	
	pLKO vs. pLKO+ Aβ_{42}	0.0454
	pLKO+ Aβ_{42} vs. miR-449a+ Aβ_{42}	0.0202
**4A**	**CDC25A WT UTR**	
	pLKO+ Aβ_{42} vs. miR-449a+ Aβ_{42}	0.0009
	**Mutant UTR**	
	pLKO+ Aβ_{42} vs. miR-449a+ Aβ_{42}	0.2835
**4B**	**Cyclin D1 WT UTR**	
	pLKO+ Aβ_{42} vs. miR-449a+ Aβ_{42}	0.0115
	**Mutant UTR**	
	pLKO+ Aβ_{42} vs. miR-449a+ Aβ_{42}	0.9999
**4C**	Ctrl vs. anti-449a	0.0065
**4D**	Ctrl vs. anti-449a	0.0108
**4E**	WT+plko vs. Tg+plko	0.0124
	Tg+plko vs. Tg+miR449a	0.0020
**4F**	WT+plko vs. Tg+plko	0.0001
	Tg+plko vs. Tg+miR449a	0.0003
**4G**	**PCNA**	
	Ctrl vs. Aβ_{42}	0.0001
	Aβ_{42} vs. Aβ_{42}+ miR-449a	0.0002
	Aβ_{42}+miR-449a vs. Aβ_{42}+449a+CDC25A	0.0356
	**Cl_caspase3**	

	Ctrl vs. $A\beta_{42}$	0.0001
	$A\beta_{42}$ vs. $A\beta_{42}$+ miR-449a	0.0001
	$A\beta_{42}$+miR-449a vs. $A\beta_{42}$+449a+CDC25A	0.0013
**4H**	**PCNA**	
	Ctrl vs. $A\beta_{42}$	0.0015
	$A\beta_{42}$ vs. $A\beta_{42}$+ miR-449a	0.0036
	$A\beta_{42}$+miR-449a vs. $A\beta_{42}$+449a+CyclinD1	0.0058
	**Cl_caspase3**	
	Ctrl vs. $A\beta_{42}$	0.0377
	$A\beta_{42}$ vs. $A\beta_{42}$+ miR-449a	0.0425
	$A\beta_{42}$+miR-449a vs. $A\beta_{42}$+449a+cyclinD1	0.0120
**5B**	WT vs. Tg	0.0023
	WT pLKO vs. Tg pLKO	0.0318
	Tg pLKO vs. Tg miR-449a	0.0196
**5D**	WT vs. Tg	0.0026
	WT pLKO vs. Tg pLKO	0.0101
	Tg pLKO vs. Tg miR-449a	0.0036
**5E**	WT vs. Tg	0.0293
	WT pLKO vs. Tg pLKO	0.0266
	Tg pLKO vs. Tg miR-449a	0.0176

Supplementary Excel files: Dataset S1-S4

Dataset S1 RNASeq Differential expression results: Excel file containing small RNA sequencing differential expression results that are represented in heat map in Fig. 1A and fold change in miRNA in TgAD with respect to WT arranged according to different fold change cutoffs.

Dataset S2 DeSeq2 normalized counts: After DESeq2 analysis, normalized counts of all the miRNA in two replicates were obtained.

Dataset S3 (For Fig 1C and S1B): Excel file with the list of miRNAs identified in other studies and in the present study that were used for generating Venn diagram in Fig. 1C and S1B.

Dataset S4 (For Fig. S2A): Cell cycle related targets of miR-449a predicted using miRDB, TargetScan and RNA22.

References

1. Modi, P. K., Jaiswal, S., and Sharma, P. (2016) Regulation of Neuronal Cell Cycle and Apoptosis by MicroRNA 34a. *Mol Cell Biol* **36**, 84-94
2. Modi, P. K., Komaravelli, N., Singh, N., and Sharma, P. (2012) Interplay between MEK-ERK signaling, cyclin D1, and cyclin-dependent kinase 5 regulates cell cycle reentry and apoptosis of neurons. *Mol Biol Cell* **23**, 3722-3730
3. Pan, R. Y., Ma, J., Kong, X. X., Wang, X. F., Li, S. S., Qi, X. L., Yan, Y. H., Cheng, J., Liu, Q., Jin, W., Tan, C. H., and Yuan, Z. (2019) Sodium rutin ameliorates Alzheimer's disease-like pathology by enhancing microglial amyloid-beta clearance. *Sci Adv* **5**, eaau6328
4. Harach, T., Marungruang, N., Duthilleul, N., Cheatham, V., Mc Coy, K. D., Frisoni, G., Neher, J. J., Fak, F., Jucker, M., Lasser, T., and Bolmont, T. (2017) Reduction of Abeta amyloid pathology in APPPS1 transgenic mice in the absence of gut microbiota. *Sci Rep* **7**, 41802
5. Howell, M. D., Bailey, L. A., Cozart, M. A., Gannon, B. M., and Gottschall, P. E. (2015) Hippocampal administration of chondroitinase ABC increases plaque-adjacent synaptic

marker and diminishes amyloid burden in aged APPswe/PS1dE9 mice. *Acta Neuropathol Commun* **3**, 54

6. Moreno-Jimenez, E. P., Flor-Garcia, M., Terreros-Roncal, J., Rabano, A., Cafini, F., Pallas-Bazarra, N., Avila, J., and Llorens-Martin, M. (2019) Adult hippocampal neurogenesis is abundant in neurologically healthy subjects and drops sharply in patients with Alzheimer's disease. *Nat Med* **25**, 554-560

A

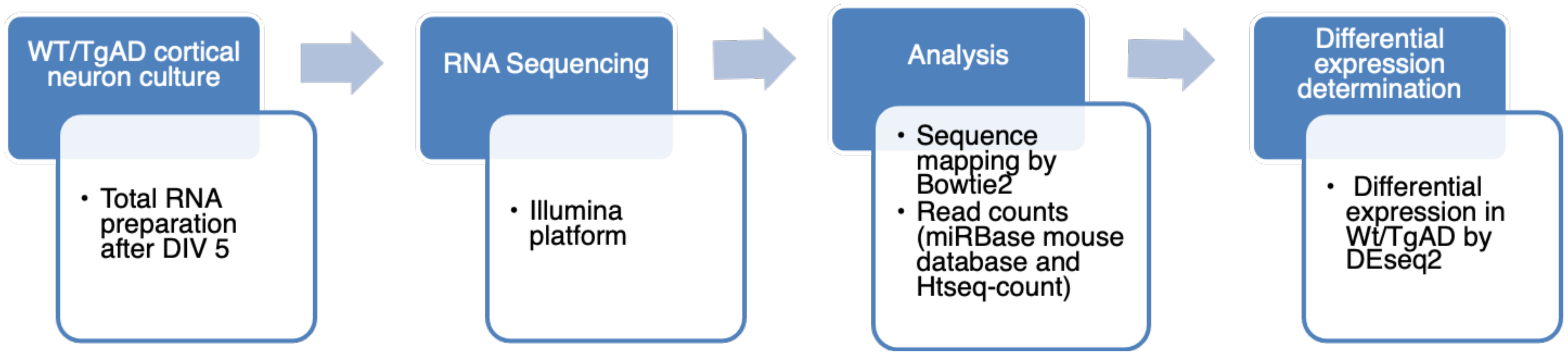

B

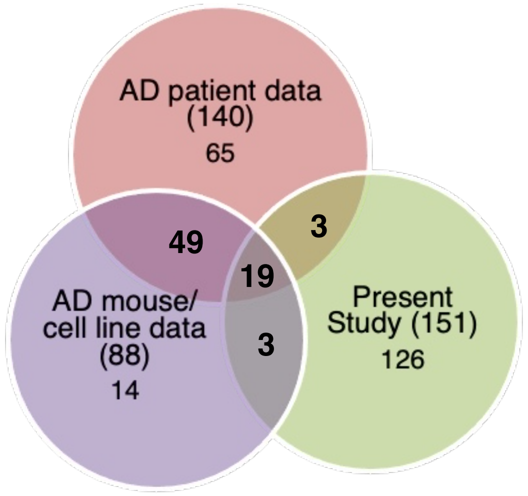

Common miRNA among three subsets

1. miR-190a-5p
2. miR-3068-3p
3. miR-449a-5p
4. miR-128-3p
5. miR-30b-5p
6. miR-34c-5p
7. miR-16-5p
8. miR-29c-3p
9. miR-99a-3p
10. miR-204-5p
11. miR-1298-5p
12. miR-30e-5p
13. miR-301a-3p
14. miR-369-3p
15. miR-744-5p
16. miR-339-3p
17. miR-1983
18. miR-149-5p
19. miR-101c

C

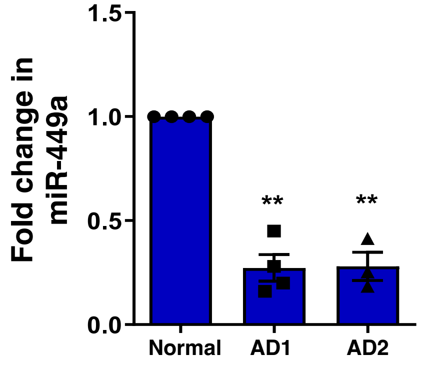

Supp. Figure S1

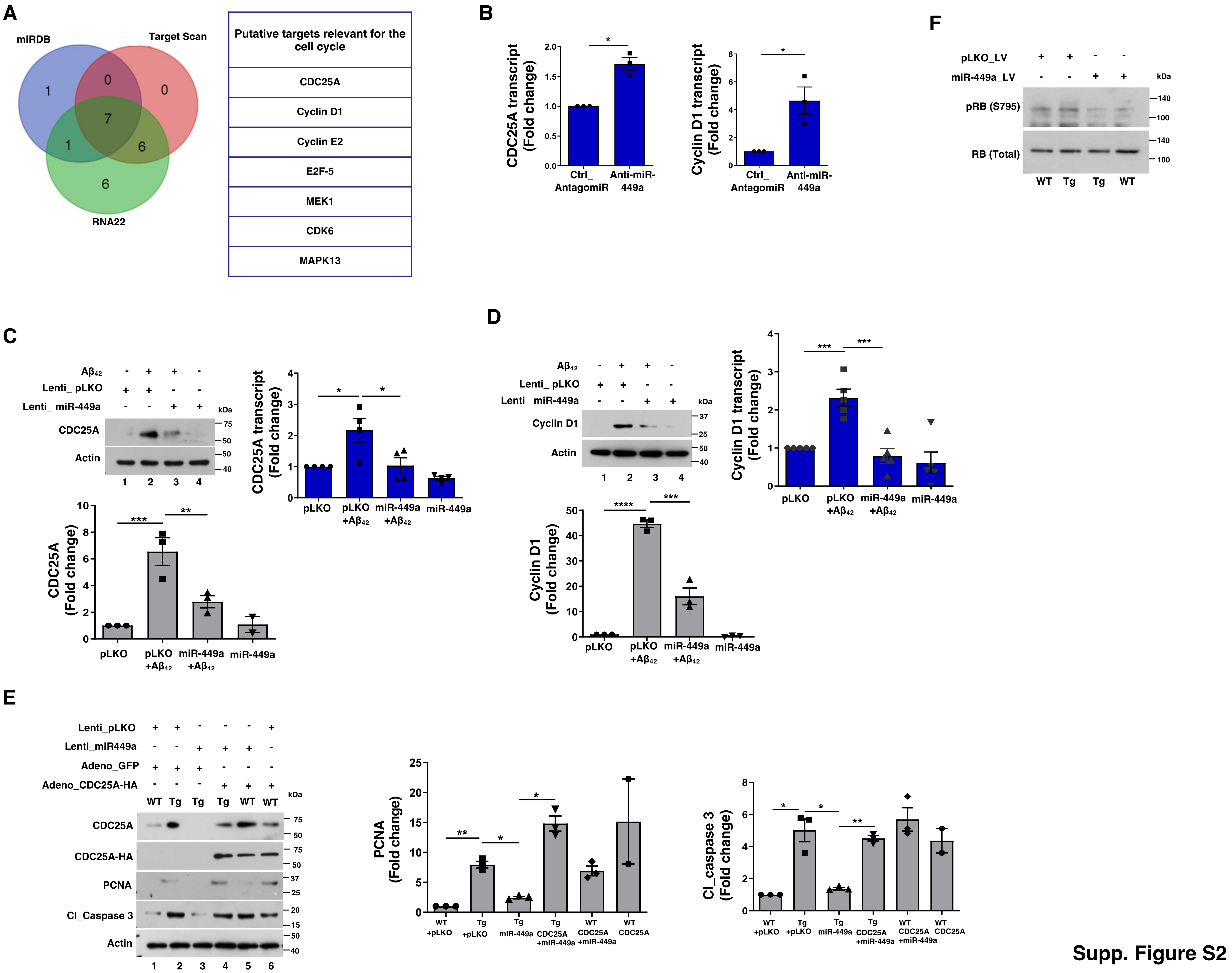

Supp. Figure S2

A

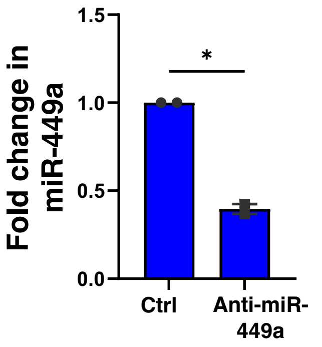

B

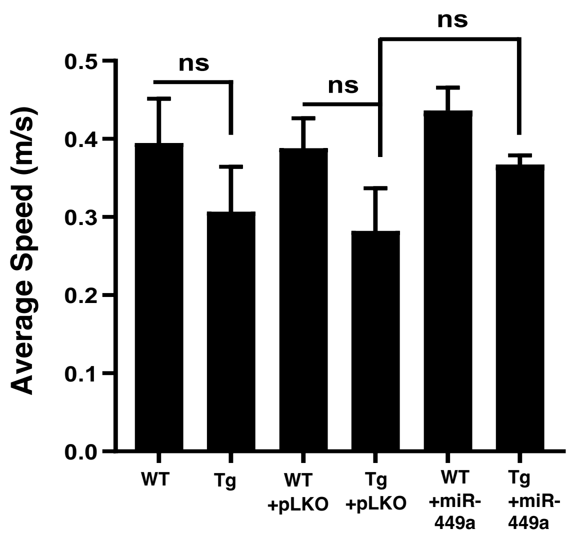

C

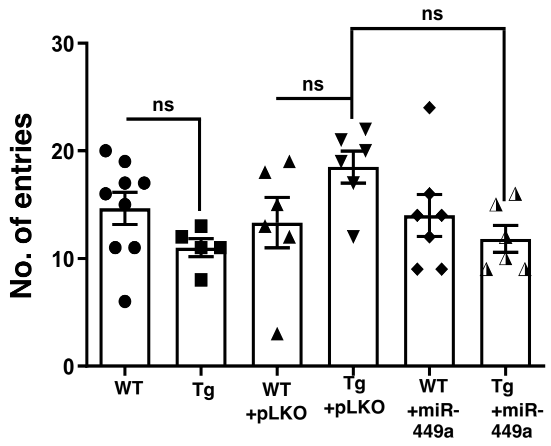

Supp. Figure S3

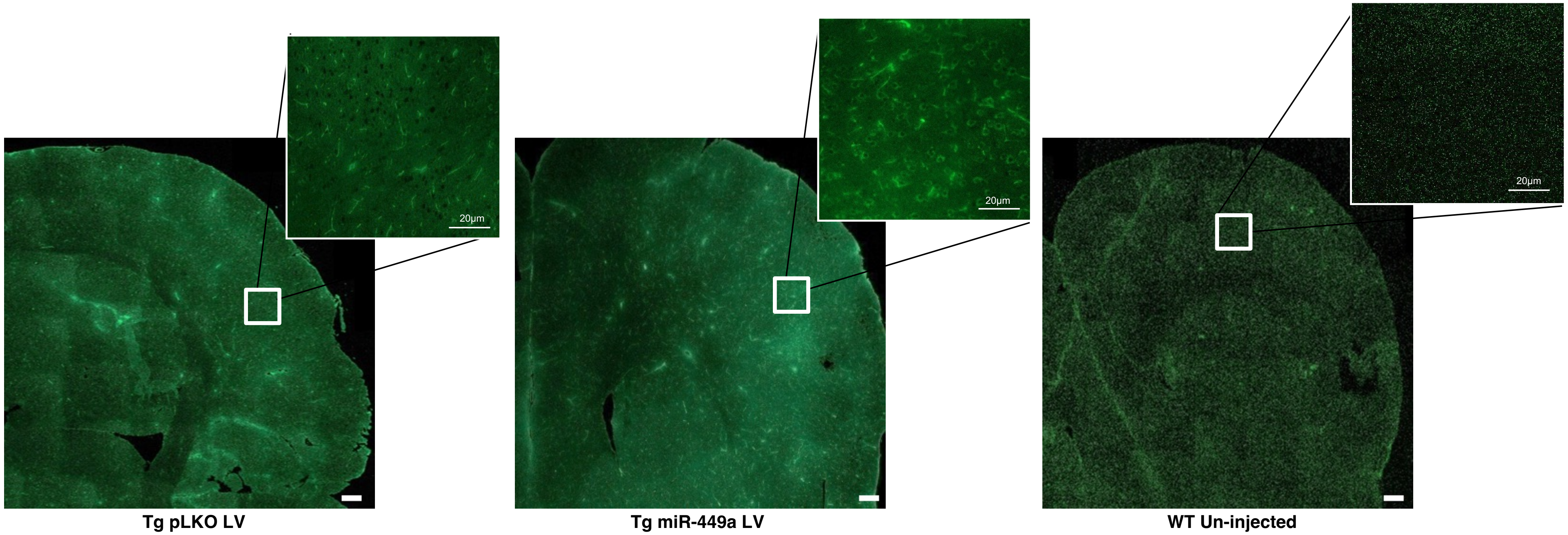

Supp. Figure S4

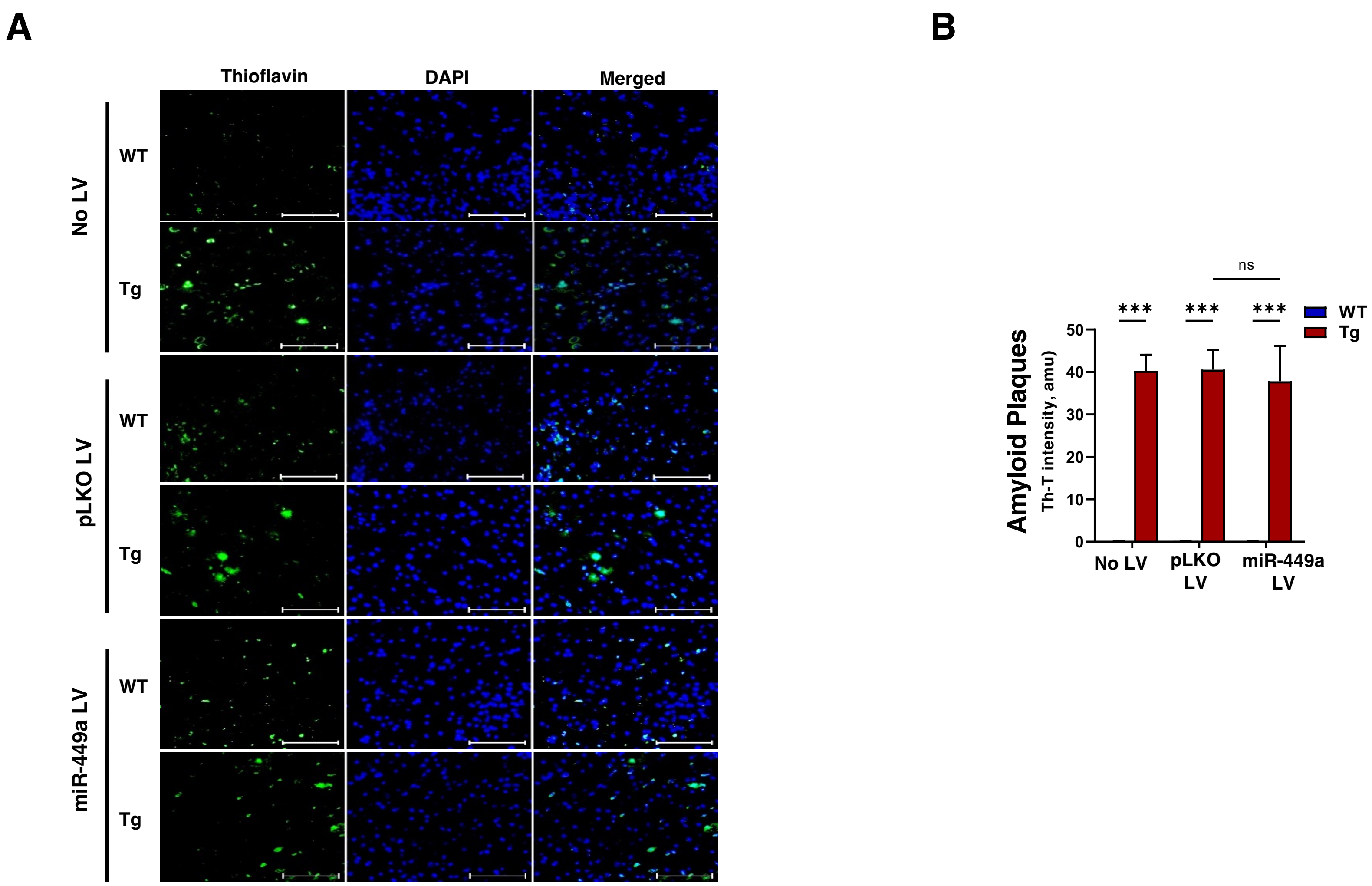

Supp. Figure S5

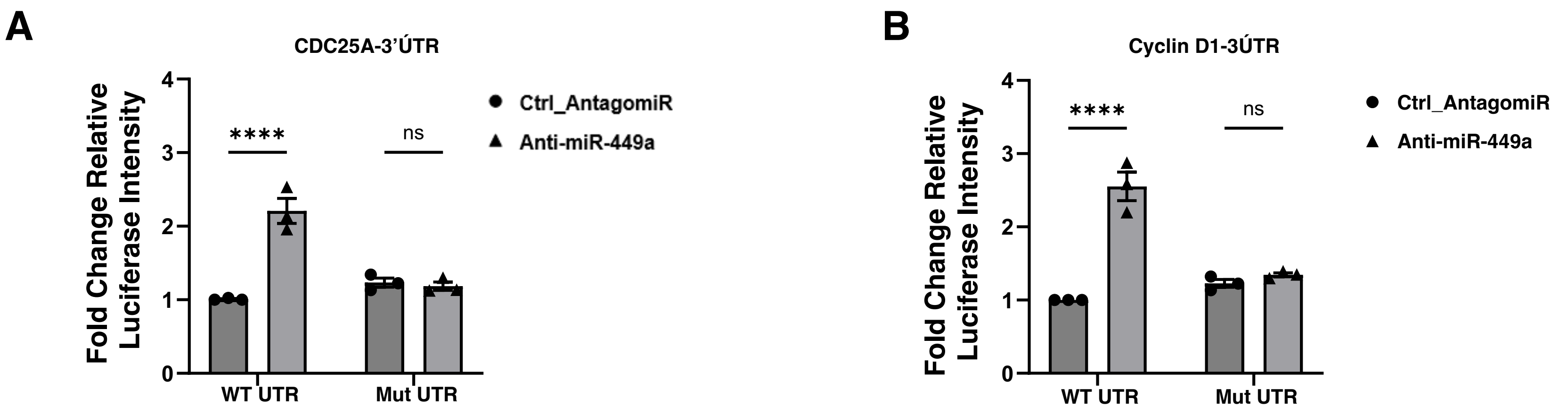

Supp. Figure S6
